# Association of *PTH1R* Gene Polymorphisms with Three-Dimensional Mandibular Morphology Across Three Recruitment Cohorts

**DOI:** 10.3390/ijms27198894

**Published:** 2026-10-07

**Authors:** Dongsan Shin, Ryosuke Kimura, Yong-Il Kim, Mohamed Adel, Seishiro Ueda, Masahiro Takahashi, Yu Hikita, Tetsutaro Yamaguchi

**Affiliations:** 1 Department of Orthodontics, School of Dentistry, Kanagawa Dental University, Yokosuka 238-8580, Japan; shidond15@gmail.com (D.S.); s.ueda@kdu.ac.jp (S.U.); takahashi.masahiro@kdu.ac.jp (M.T.); hickyyu@gmail.com (Y.H.); 2 Department of Human Biology and Anatomy, Graduate School of Medicine, University of the Ryukyus, Ginowan 903-2720, Japan; rkimura@cs.u-ryukyu.ac.jp; 3 Department of Orthodontics, School of Dentistry, Pusan National University, Yangsan 50612, Republic of Korea; kimyongil@pusan.ac.kr; 4 Department of Orthodontics, College of Dentistry, Texas A&M University, Dallas, TX 75246, USA; mohadel@tamu.edu

**Keywords:** *PTH1R*, mandibular morphology, single-nucleotide polymorphism, geometric morphometrics

## Abstract

Parathyroid hormone 1 receptor (PTH1R) regulates chondrocyte differentiation and mandibular condylar cartilage development, but its contribution to adult mandibular morphology remains unclear. We investigated associations of two *PTH1R* single-nucleotide polymorphisms (SNPs), rs724448 and rs2242116, with three-dimensional mandibular morphology in 448 orthodontic patients recruited in Japan (199), Korea (79), and Egypt (170). Salivary DNA was genotyped, and computed tomography images were analyzed using geometric morphometrics and conventional measurements. Multivariate Procrustes analysis evaluated overall mandibular shape. Cohort-specific regressions examined natural-log-transformed centroid size (ln(centroid size)), principal component scores, and conventional measurements, adjusting for sex and age and additionally for imaging modality in Japan. Estimates were combined using inverse-variance fixed-effect meta-analysis. Neither variant showed a significant whole-shape association after correction for two SNP tests. Secondary analyses yielded negative estimates for ln(centroid size) and nominal associations with selected anteroposterior dimensions and cohort-specific principal component scores. None remained significant after either Bonferroni or Benjamini–Hochberg false-discovery-rate correction across 408 tests. These findings provide no statistically robust evidence linking either variant to adult mandibular morphology. Nominal findings require independent replication.

## 1. Introduction

Facial morphology comprises multiple complex traits, each of which is influenced by genetic and environmental factors [1]. Cephalometric twin studies have demonstrated genetic influences on craniofacial morphology [2], and mandibular morphology has a substantial heritable component [3].

The parathyroid hormone 1 receptor (PTH1R) is activated by parathyroid hormone (PTH) and parathyroid hormone-related protein (PTHrP) and plays an important role in regulating skeletal development, chondrocyte differentiation, and bone metabolism [4,5,6]. In particular, PTHrP and Indian hedgehog (Ihh) participate in a feedback loop that regulates growth plate chondrocyte differentiation [7]. PTHrP–PTH1R signaling also contributes to chondrocyte differentiation and development of the mandibular condylar cartilage [8,9]. Rare pathogenic variants in *PTH1R* can cause primary failure of eruption (PFE) and skeletal disorders [10,11,12,13,14]. Craniofacial abnormalities have also been described in Jansen metaphyseal chondrodysplasia (JMC), which results from activating *PTH1R* variants [15,16,17,18]. These observations establish the biological relevance of PTH1R signaling but do not demonstrate that common polymorphisms influence adult mandibular morphology.

A previous candidate-gene study reported nominal haplotype-level associations between a region spanning single-nucleotide polymorphisms (SNPs) rs724448 and rs2242116 and bone mineral density, although no associations were detected for the individual *PTH1R* SNPs [19]. In addition, rs2242116 was identified as an SNP associated with birth weight in a large-scale genome-wide association study (GWAS) [20,21]. Birth weight is an indicator of fetal growth and exhibits genome-wide genetic correlations with birth length and adult height [20,21].

When evaluating genetic associations across recruitment cohorts, population-related differences in allele frequencies and linkage disequilibrium (LD) structures should be considered [22,23,24]. Differences in allele frequencies may affect statistical power and the precision of effect estimates, whereas differences in LD patterns may influence observed associations when the genotyped SNPs tag causal variants differently across cohorts [23,24]. Accordingly, investigating associations between genetic polymorphisms and mandibular morphological variation across clinic-based cohorts recruited in different geographic settings may provide insight into the consistency of observed associations across cohorts [25]. However, more studies are needed in this area. This study aimed to quantify three-dimensional mandibular morphology using geometric morphometrics and principal component analysis (PCA) of computed tomography (CT) data from orthodontic patients recruited in Japan, Korea, and Egypt, and to assess its associations with the *PTH1R* SNPs rs724448 and rs2242116.

This study was designed to evaluate whether the two candidate *PTH1R* variants show consistent associations with mandibular morphology across geographically distinct recruitment cohorts.

## 2. Results

The final analytical sample comprised 448 orthodontic patients recruited in Japan (*n* = 199), Korea (*n* = 79), and Egypt (*n* = 170). Demographic characteristics, imaging modalities, skeletal classifications, and cephalometric measurements are summarized in Table 1. Based on the A point–nasion–B point (ANB) angle, 183 participants (40.8%) were classified as skeletal Class I, 194 (43.3%) as Class II, and 71 (15.8%) as Class III. All CT images had originally been acquired for clinical orthodontic diagnosis and treatment planning. The final genotyping call rate was 97.8% for rs724448 and 100% for rs2242116. The 10 samples with unresolved rs724448 calls showed low fluorescence signals in both allele channels and remained uncalled after repeat genotyping. Allelic discrimination plots of the initial and repeat runs containing these samples are provided in Figure S2. Technical duplicate genotyping of 20 samples showed 100% concordance for both SNPs. Neither SNP showed a statistically significant deviation from Hardy–Weinberg equilibrium in any cohort (all exact *p* values ≥ 0.152). Cohort-specific call rates and repeat-testing results are presented in Table S1A, and genotype counts, minor allele frequencies, and Hardy–Weinberg equilibrium results are presented in Table S1B.

**Figure 1:**
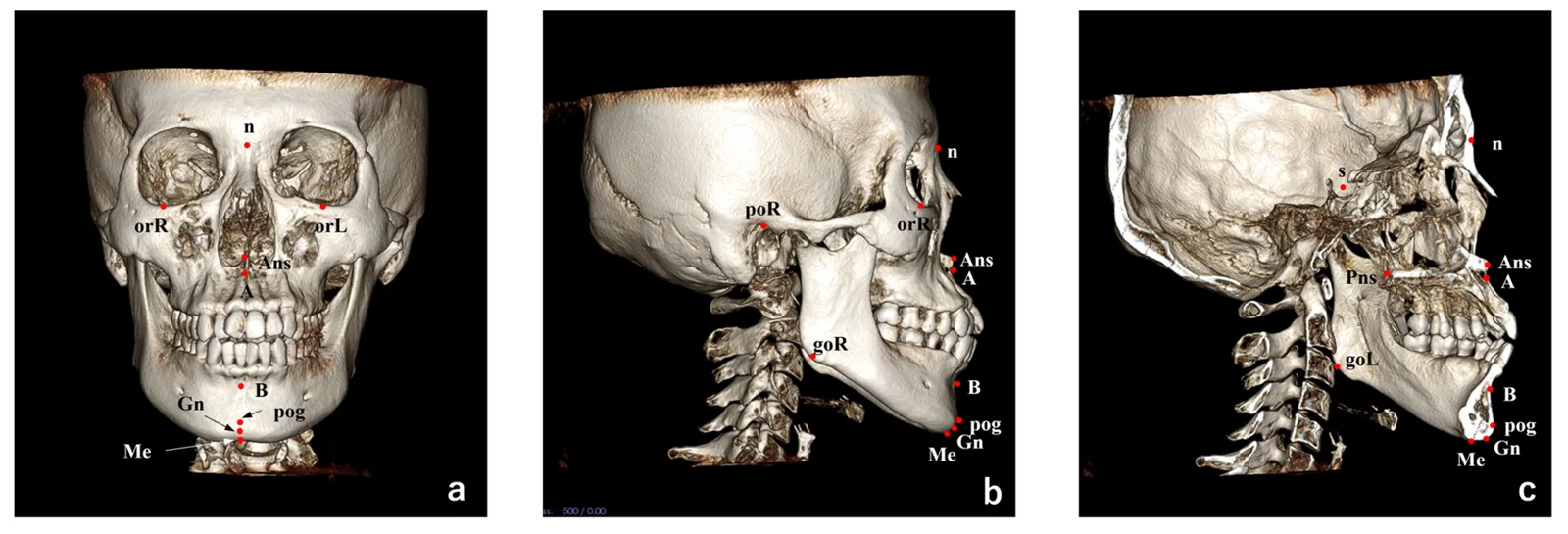
Landmarks used for craniofacial measurement. (**a**) Frontal view. (**b**) Lateral view. (**c**) Clipping-lateral view. n, nasion; Or, orbitale R/L; Po, porion R/L; S, sella; A, A point; B, B point; Pog, pogonion; Gn, gnathion; Me, menton; Go, gonion R/L; ANS, anterior nasal spine; PNS, posterior nasal spine; R/L, right/left.

**Figure 2:**
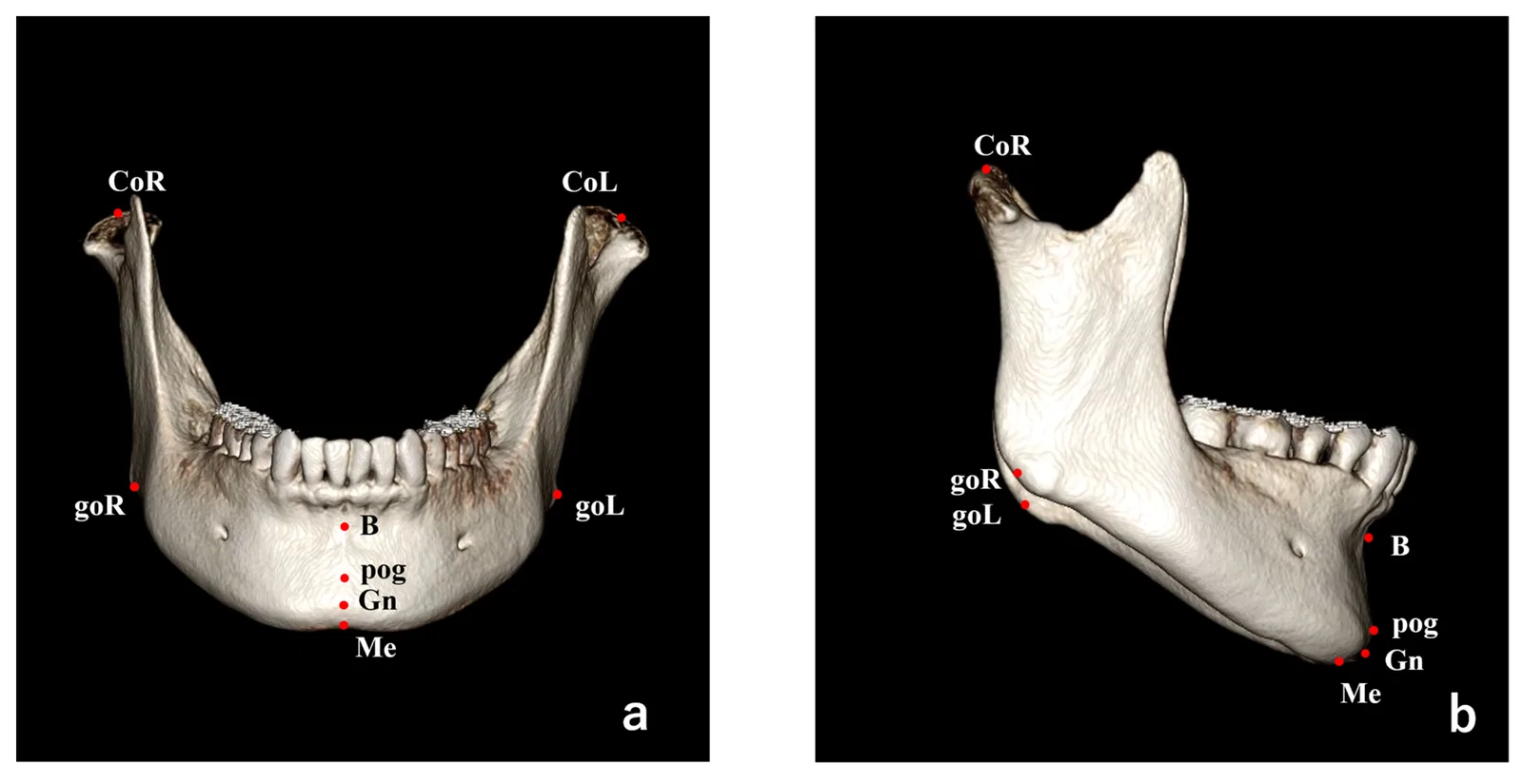
Landmarks used for mandibular measurement. (**a**) Frontal view. (**b**) Lateral view. Co, condylion R/L; B, B point; pog, pogonion; Gn, gnathion; Me, menton; go, gonion R/L; R/L, right/left.

**Figure 3:**
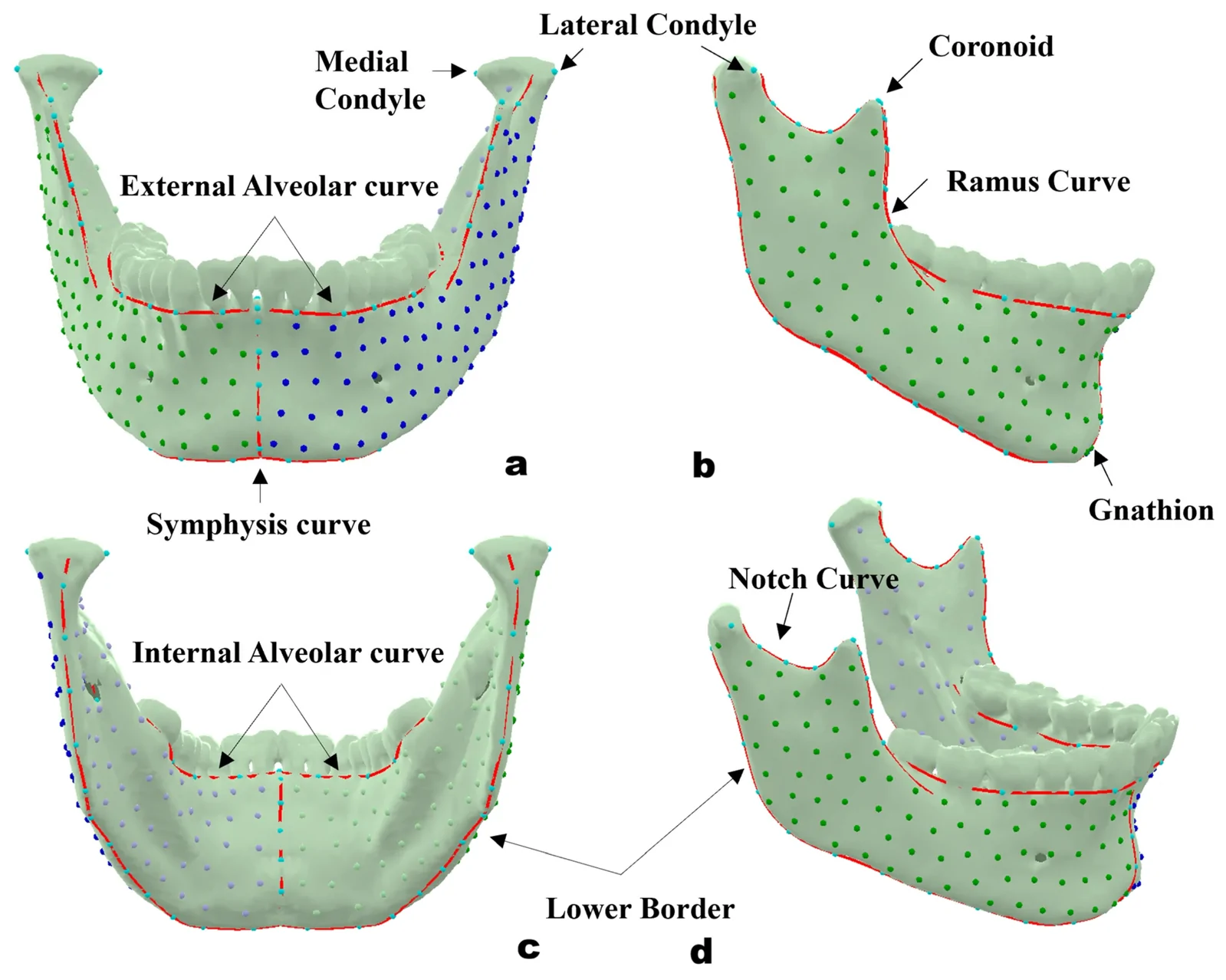
Configuration of fixed landmarks, curve semi-landmarks, and surface semi-landmarks on the mandibular mesh. (**a**) Anterior view of the external surface of the mandible. (**b**) Lateral view of the external surface of the mandible. (**c**) Posterior view of the internal surface of the mandible. (**d**) Posterolateral view of the internal surface of the mandible. The Gnathion helper point was used only to facilitate semi-landmark placement and was excluded from generalized Procrustes analysis (GPA), principal component analysis (PCA), and centroid size calculation. Light blue points represent fixed landmarks and curve sliding semi-landmarks. Red dotted lines indicate the curves along which the curve sliding semi-landmarks are positioned. Surface semi-landmarks are color-coded as follows: green, right external mandibular surface; blue, left external mandibular surface; light green, right internal mandibular surface; and light purple, left internal mandibular surface.

Figure 1, Figure 2 and Figure 3 show mandibular morphology evaluated by placing fixed landmarks and semi-landmarks on the CT images.

Derived-allele frequencies and LD estimates are presented in Table 2. The r^2^ values between rs724448 and rs2242116 were 0.95, 1.00, and 0.67 in the Japan-, Korea-, and Egypt-recruited cohorts, respectively. In the Korea-recruited cohort, the two SNPs had identical derived-allele dosage variables and therefore yielded identical regression coefficients, standard errors, and *p* values; these results should not be interpreted as independent association signals.

The final shape configuration comprised 353 landmarks and semi-landmarks. Principal component (PC) 1 and PC2 explained 23.3% and 11.8% of total shape variation, respectively; PC1–PC5 together explained 53.9%, and PC1–PC11 explained 70.3%. Shape variation along PC1–PC11 is illustrated in Figure S1 using configurations at +3 standard deviations (SD) and −3 SD from the mean. The independent R version 4.5.1 (R Foundation for Statistical Computing, Vienna, Austria) implementation closely reproduced the Viewbox-derived natural-log-transformed centroid size and PC scores, with correlations of at least 0.9995 after alignment of PC-axis signs. The visualizations in Figure S1 illustrate variation in overall anteroposterior mandibular configuration (PC1), ramus height and mandibular body breadth (PC2), anteroposterior and vertical dimensions (PC3 and PC9), and mandibular length and body depth (PC7 and PC8). PC10 and PC11 were retained as higher-order axes of shape variation without assigning specific anatomical interpretations. Anatomical interpretation of PC4, PC5, PC6, PC10, and PC11 was limited by their low repeatability (intraclass correlation coefficient (ICC) range, 0.30–0.59; see Table S6); these axes are therefore displayed descriptively in Figure S1 but are not used to support anatomical or biological conclusions.

In 30 participants with repeated digitizations, intra-observer landmark-placement errors ranged from 0.12 to 0.63 mm. The ICC for natural-log-transformed centroid size was 0.99 (95% confidence interval (CI), 0.94–0.99). ICC estimates were 0.96 for PC1 and 0.91 for PC2 and ranged from 0.30 to 0.86 for PC3–PC11. Repeatability was limited for PC4, PC5, PC6, PC10, and PC11, with ICC estimates ranging from 0.30 to 0.59 (Table S6). Whole-shape repeatability across the 30 paired digitizations was R = 0.504 (95% bootstrap CI, 0.390–0.592). Within-participant variation accounted for 25.42% of the total Procrustes sum of squares in the repeated-digitization dataset (Table S6). These repeatability analyses evaluated landmark digitization and subsequent geometric morphometric processing and should not be interpreted as direct validation of segmentation accuracy or cross-modality comparability.

In the primary whole-shape Procrustes analysis of variance (ANOVA), neither rs724448 (R^2^ = 0.23%, F = 1.14, permutation *p* = 0.28; Bonferroni-adjusted *p* = 0.55) nor rs2242116 (R^2^ = 0.21%, F = 1.08, permutation *p* = 0.33; Bonferroni-adjusted *p* = 0.66) showed a statistically significant association with overall mandibular shape. In the two pooled primary models, approximately 8.5% and 1.2% of total shape variance were associated with the recruitment-cohort and imaging-modality terms, respectively. Because scanner systems were specific to recruitment cohorts, the variance associated with the cohort term could not be separated from scanner-related variation and should not be interpreted as a purely biological difference between cohorts. SNP-by-cohort interaction tests were also not statistically significant (Bonferroni-adjusted *p* = 0.75 for rs724448 and 0.53 for rs2242116; see Table 3).

Under the approximate regression-based sensitivity analysis, the minimum detectable partial R^2^ at 80% power and α = 0.025 was 2.09% for the pooled sample (*n* = 448), 4.62% for Japan (*n* = 199), 11.07% for Korea (*n* = 79), and 5.37% for Egypt (*n* = 170). These values are illustrative benchmarks and are not directly equivalent to the total-shape R^2^ reported for the Procrustes analyses.

Of the 408 secondary univariate association tests, 23 (5.6%) yielded unadjusted *p* values below 0.05. None remained statistically significant after Bonferroni or Benjamini–Hochberg false-discovery-rate (BH-FDR) correction (Table S7). All *p* values reported below are unadjusted. In the inverse-variance fixed-effect meta-analysis, both SNPs showed nominal negative associations with natural-log-transformed centroid size: rs724448 (B = −0.0053, *p* = 0.023) and rs2242116 (B = −0.0054, *p* = 0.019). These estimates correspond to approximately 0.53% and 0.54% smaller centroid size, respectively, per additional derived allele (Table 4). In the cohort-specific analyses, rs724448 also showed a nominal negative association with natural-log-transformed centroid size in the Egypt-recruited cohort (B = −0.0084, *p* = 0.029). Nominal associations with PC scores were observed for rs2242116 with PC5 in the Japan-recruited cohort, both SNPs with PC3 and PC9 in the Korea-recruited cohort, and both SNPs with PC8 in the Egypt-recruited cohort (Table 4). A descriptive visualization of shape variation along PC8 is provided in Figure S3. These within-cohort findings do not establish differences in genetic effects between cohorts; formal heterogeneity analyses are reported separately. For conventional measurements, the fixed-effect meta-analysis showed nominal negative associations of rs724448 with mandibular body length, mid-Co–B, and mid-Co–Gn (Table S2). In the Egypt-recruited cohort, rs724448 showed nominal negative associations with sella–nasion (SN) length, mandibular body length, ANS–PNS, intergonial width (Go–Go), mid-Co–B, mid-Co–Gn, ANS–PNS/Co–Co, and Go–Go/Co–Co. Rs2242116 showed nominal negative associations with intergonial width and Go–Go/Co–Co in the same cohort (Table S2). None of these nominal associations remained statistically significant after multiple-testing correction.

Among the 199 participants in the Japan-recruited cohort, 158 (79.4%) were examined using cone-beam computed tomography (CBCT) and 41 (20.6%) using multidetector computed tomography (MDCT). No statistically significant difference in genotype distribution was detected between imaging modalities for either rs724448 (Fisher’s exact *p* = 0.97) or rs2242116 (Fisher’s exact *p* = 1.0) (Table S3). In the modality-adjusted analyses of the full Japan-recruited cohort, rs2242116 showed a nominal association with PC5 (unadjusted *p* = 0.031), whereas no nominal associations were observed for rs724448. In the CBCT-only analyses, nominal associations were observed with PC5 and PC7 for rs724448 (unadjusted *p* = 0.036 and 0.0056, respectively) and rs2242116 (unadjusted *p* = 0.020 and 0.010, respectively) (Table S4). In the Japan CBCT-only whole-shape sensitivity analysis, neither rs724448 nor rs2242116 showed a statistically significant association with overall mandibular shape (Bonferroni-adjusted *p* = 0.98 and 0.44, respectively; see Table 3). Thus, the CBCT-only whole-shape analysis also yielded no statistically significant genotype association. In the rs2242116 sensitivity analysis including all 458 eligible participants, the fixed-effect meta-analysis results were broadly similar to those obtained in the original 448-participant sample (Table S8). For ln(centroid size), the estimate was −0.0052 (95% CI, −0.0097 to −0.0007; unadjusted *p* = 0.022), compared with −0.0054 (95% CI, −0.0100 to −0.0009; unadjusted *p* = 0.019) in the original sample. No association remained statistically significant after Bonferroni or BH-FDR correction across the 204 sensitivity tests. Thus, inclusion of the 10 additional participants did not change the multiple-testing-adjusted conclusion for the secondary rs2242116 analyses. Between-cohort heterogeneity was evaluated across 102 SNP–outcome combinations. Seven combinations showed nominal evidence of heterogeneity: PC3, PC9, and intergonial width for both SNPs, and ANS–PNS for rs724448. None remained statistically significant after Bonferroni or BH-FDR correction across the 102 heterogeneity tests. Nevertheless, I^2^ estimates were approximately 78–82% for PC3, 75% for PC9, 72–73% for intergonial width, and 61–64% for PC8, indicating that the absence of corrected statistical significance should not be interpreted as evidence of homogeneous effects. For rs724448, between-cohort heterogeneity was not statistically significant for mandibular body length, mid-Co–B, mid-Co–Gn, or mid-Go–Gn (Table S5). Similarly, the heterogeneity tests for PC8 were not statistically significant for either rs724448 (Pheterogeneity = 0.078) or rs2242116 (Pheterogeneity = 0.063). DerSimonian–Laird random-effects estimates are presented as sensitivity analyses in Table S5 to assess the sensitivity of the meta-analysis results to the modeling of between-cohort heterogeneity.

## 3. Discussion

The primary whole-shape Procrustes ANOVA did not identify a statistically significant association between either *PTH1R* variant and overall mandibular shape. None of the 408 secondary univariate associations remained statistically significant after Bonferroni or BH-FDR correction. Nominal associations involving natural-log-transformed centroid size, selected mandibular length measurements, and cohort-specific PC scores should therefore be interpreted as exploratory findings requiring independent replication [26]. These results do not establish an association of either variant with mandibular size or shape, although the limited sample size means that small effects cannot be excluded [27]. Although the secondary analyses yielded nominal negative estimates for natural-log-transformed centroid size and several conventional measurements, these findings were not consistently observed across cohorts. The nominal conventional-measurement associations were concentrated mainly in the Egypt-recruited cohort and involved several correlated anteroposterior and transverse dimensions. Moreover, none was supported by the multiple-testing-adjusted analyses or the primary whole-shape analysis. These patterns therefore do not define a reproducible mandibular phenotype associated with either variant (Table 4 and Table S2).

The proportion of nominally significant secondary tests (23/408, 5.6%) was close to the 5% expected under the global null. Because the SNPs, outcomes, and analysis sets are correlated, this comparison is descriptive rather than a formal test of enrichment. Together with the absence of associations surviving multiple-testing correction, this observation reinforces the need for caution when interpreting individual nominal findings.

The Genotype-Tissue Expression (GTEx) project provides a resource for investigating tissue-specific genetic regulation of gene expression, as described in the GTEx Consortium’s V8 atlas [28]. The expression quantitative trait locus (eQTL) annotations retrieved from GTEx Analysis Release V10 showed associations of the alternative alleles (ALT) of rs724448 and rs2242116 with lower *PTH1R* expression in adipose and breast tissues (Table 5). These associations provide indirect functional annotation, but their relevance to mandibular bone or condylar cartilage remains unknown. After harmonizing the effect alleles to A for both SNPs, the A alleles were associated with higher *PTH1R* expression in the adipose and breast tissues shown in Table 5 and with nominally smaller centroid size in the fixed-effect meta-analysis. The centroid-size associations did not remain significant after multiple-testing correction. These findings provide indirect functional context but do not establish that increased *PTH1R* expression causes reduced mandibular size, particularly because the eQTL results were obtained from tissues other than mandibular bone or condylar cartilage.

Likewise, craniofacial manifestations of Jansen metaphyseal chondrodysplasia provide biological context [15,16,17,18], but effects of rare activating *PTH1R* variants cannot be directly extrapolated to these common polymorphisms. Functional studies in relevant mandibular tissues are needed [29,30].

The similar estimates for rs724448 and rs2242116 should be interpreted in the context of their high linkage disequilibrium (Table 2) [31]. In the Korea-recruited cohort, the two SNPs had identical derived-allele dosages and consequently yielded identical association estimates. These results represent redundant genotype information and do not provide independent replication. Correlation between the two variants can also produce similar nominal association patterns, including patterns arising through sampling variation; the present findings do not identify a causal variant [32,33,34].

Differences in nominal significance among cohorts do not establish differences in genetic effects. Neither the corrected heterogeneity tests nor the SNP-by-cohort interaction tests provided statistically significant evidence of between-cohort differences. Nevertheless, the relatively high I^2^ estimates for several outcomes indicate that homogeneous effects cannot be assumed [35]. With only three cohorts, heterogeneity estimates are imprecise, and both the cohort-specific estimates and the fixed-effect summaries require cautious interpretation [36]. The DerSimonian–Laird random-effects estimates in Table S5 provide a sensitivity analysis of the dependence of the summary results on the meta-analytic model [37].

A strength of this study is the combination of whole-shape geometric morphometrics and conventional craniofacial measurements across three recruitment cohorts. The use of a multivariate primary analysis, explicit genotyping quality control, and imaging-modality sensitivity analyses supports a more transparent assessment of the candidate-variant associations.

Several limitations should be considered. Participants were recruited from orthodontic clinics and underwent imaging for clinical indications, which limits generalizability to the broader adult population. Participants classified as skeletal Class I were also orthodontic patients and did not constitute a separately recruited unaffected control group. Previous orthognathic surgery, mandibular trauma, relevant pathology, and the extent of tooth loss were not uniformly documented, preventing their application as standardized exclusion criteria.

The sample sizes, particularly that of the Korea-recruited cohort, limited the precision of effect estimates and the ability to detect small associations [27]. The approximate sensitivity analysis indicated detectable partial R^2^ values of approximately 2.1% in the pooled sample and 11.1% in the Korea-recruited cohort at 80% power and α = 0.025. These regression-based benchmarks are not directly equivalent to the observed whole-shape R^2^ values of 0.21–0.23% and should not be interpreted as exact detection limits for the multivariate analysis. Nevertheless, the study provides limited information about small genetic effects, and the nonsignificant findings should not be interpreted as evidence against effects of the magnitude plausibly expected for common variants influencing a complex morphological trait.

Cohort membership was based on recorded nationality and recruitment setting rather than genome-wide genetic ancestry. Adjustment for recruitment cohort in the primary analysis and stratification by cohort in the secondary analyses could not address unmeasured genetic substructure within cohorts [38]. Furthermore, the two candidate SNPs provided limited coverage of the *PTH1R* locus and did not capture the broader genetic and environmental contributions to mandibular morphology [39,40].

Scanner systems and acquisition protocols differed by recruitment cohort, and no scanner system was shared across cohorts. Consequently, the approximately 8.5% of total shape variance associated with the recruitment-cohort term in Table 3B could not be separated into biological cohort differences and scanner-related effects. Adjustment for CBCT versus MDCT and the Japan CBCT-only sensitivity analyses did not resolve this confounding or establish comparability across scanners.

Repeated digitization evaluated landmark placement and subsequent geometric morphometric processing on existing meshes, rather than independent repetition of segmentation. Whole-shape repeatability was limited (R = 0.504; 95% bootstrap CI, 0.390–0.592), indicating appreciable within-participant variation across repeated digitizations. This measurement variability may have reduced sensitivity to small genotype-related shape differences. Accordingly, the absence of statistically significant whole-shape associations should not be interpreted as evidence that small effects are absent. Repeatability was also limited for PC4, PC5, PC6, PC10, and PC11. The limited repeatability of PC5 is particularly relevant to its nominal association in the Japan-recruited cohort, which should therefore be interpreted cautiously (Table S6).

Although generalized Procrustes analysis removed differences in uniform scale and natural-log-transformed centroid size was analyzed separately, no additional allometric adjustment of the Procrustes shape coordinates for centroid size was performed. Therefore, allometric (size-related) and non-allometric (size-independent) components of mandibular shape variation were not evaluated separately [41]. In addition, the analyses of ln(centroid size) were not adjusted for stature. Therefore, any underlying association could reflect general body size rather than a relationship specific to mandibular size. This possibility is particularly relevant to rs2242116, given its previously reported association with birth weight [20,21], although the nominal centroid-size associations in the present study did not survive multiple-testing correction.

The higher initial no-call rates for rs724448 in the Egypt- and Korea-recruited cohorts, despite complete initial calls for rs2242116 in the same samples, suggest that assay-specific factors may have contributed rather than a general DNA-quality problem alone. However, the available call-rate data do not establish the underlying mechanism, and genotype-dependent missingness for rs724448 cannot be excluded. Including all 458 eligible participants in the rs2242116 sensitivity analysis did not change the multiple-testing-adjusted conclusions for the secondary outcomes. This finding addresses the sensitivity of the rs2242116 results to complete-case exclusion but does not establish that the unresolved rs724448 calls were missing at random.

In conclusion, the primary whole-shape analysis and the multiple-testing-adjusted secondary analyses provided no statistically robust evidence that rs724448 or rs2242116 is associated with adult mandibular morphology. However, the limited sensitivity to small effects precludes interpreting these findings as evidence that such effects are absent. The nominal associations with natural-log-transformed centroid size, selected conventional measurements, and cohort-specific PC scores should be regarded as hypothesis-generating findings. Larger independent studies incorporating genome-wide ancestry information, standardized imaging protocols, broader coverage of the *PTH1R* locus, and functional evaluation in mandibular tissues are required to determine whether common *PTH1R* variation contributes to adult mandibular morphology.

## 4. Materials and Methods

### 4.1. Data Collection

After imaging-related and other non-genetic eligibility exclusions, 458 participants were eligible for genotyping quality control: 200 recruited in Japan, 83 in Korea, and 175 in Egypt. Following genotyping quality control, 448 participants with complete genotype data for both SNPs were included in the association analyses: 199 in the Japan-recruited cohort, 79 in the Korea-recruited cohort, and 170 in the Egypt-recruited cohort. These 448 participants constituted the original complete-case analytical sample. A separate sensitivity analysis of rs2242116 included all 458 eligible participants, including the 10 participants excluded from the original sample because of unresolved rs724448 genotype calls. Cohort membership was defined according to recorded nationality and the country and institution of recruitment. Recorded nationality was the only individual-level descriptor consistently available for cohort classification; detailed information on individual or parental geographic origin and genome-wide genetic ancestry was not available. Accordingly, the cohorts should not be interpreted as genetically homogeneous ancestry groups. The Japan-recruited cohort included 53 men aged 19–56 years (mean age, 27.7 years) and 146 women aged 18–67 years (mean age, 28.6 years). The Korea-recruited cohort included 33 men aged 18–44 years (mean age, 24.5 years) and 46 women aged 18–47 years (mean age, 25.8 years). The Egypt-recruited cohort included 77 men aged 18–49 years (mean age, 24.8 years) and 93 women aged 18–50 years (mean age, 24.0 years).

All participants underwent CBCT or MDCT for diagnostic purposes at their respective orthodontic clinics. Eligibility was restricted to adults aged 18 years or older. Participants with documented congenital craniofacial anomalies, including cleft lip and palate, or systemic diseases known to affect craniofacial growth were excluded. Information regarding previous orthognathic surgery, mandibular trauma, relevant mandibular pathology, and the extent of tooth loss was not uniformly documented across the three cohorts. Temporomandibular disorder and obstructive sleep apnea status were not systematically collected across the three cohorts and could not be incorporated into the eligibility criteria or association models. These conditions could therefore not be applied as standardized exclusion criteria, and the absence of uniform ascertainment was considered a study limitation.

Sagittal skeletal patterns were classified according to the ANB angle as follows: Class I, 0° ≤ ANB ≤ 4°; Class II, ANB > 4°; and Class III, ANB < 0°. Cohort-specific skeletal class distributions and imaging characteristics are presented in Table 1 [42].

Written informed consent was obtained from all participants before their inclusion in the study. This study was conducted in accordance with the guidelines of the Declaration of Helsinki and approved by the relevant ethics committees at each participating institution: Kanagawa Dental University (No. 971), Pusan National University Dental Hospital (IRB No. PNUDH-2019-025), and Suez Canal University (IRB No. 8).

### 4.2. Genotyping

Saliva samples were collected from the participants using an Oragene DNA collection kit (DNA Genotek, Ottawa, ON, Canada), and genomic DNA was extracted from the saliva samples. Genotyping of the two *PTH1R* SNPs, rs724448 and rs2242116, was performed using a TaqMan genotyping assay (Life Technologies, Carlsbad, CA, USA) [43]. Allele frequencies were determined based on the obtained genotypes. Genotyping quality control was performed separately for each SNP and cohort. Samples that initially yielded no genotype call were subjected to repeat genotyping. SNP-specific call rates were calculated as the number of participants with a definitive genotype call divided by the number of participants eligible for genotyping after imaging-related and other non-genetic exclusions. Participants with an unresolved genotype call for either SNP were excluded from the complete-case association analyses. Genotype distributions and minor allele frequencies were calculated separately for each recruitment cohort, and Hardy–Weinberg equilibrium was assessed using a two-sided exact test [44]. Technical duplicate genotyping was performed for 20 samples, and genotype concordance was assessed for both SNPs. Call rates, repeat-testing results, technical duplicate concordance, and exclusions are reported in Table S1A. Genotype counts, minor allele frequencies, and Hardy–Weinberg equilibrium results for the final complete-case analytical sample are reported in Table S1B. LD coefficients (D′ and r^2^) were calculated using Haploview version 4.1 (Broad Institute, Cambridge, MA, USA) [45] (Table 2).

### 4.3. Craniofacial Measurements

Craniofacial data for the Japan-recruited cohort were obtained using CBCT and MDCT. CBCT images were acquired using a CBCT scanner with a voxel size of 0.3 mm (KaVo OP 3D Vision, Biberach, Germany), and MDCT images were acquired using an Aquilion Prime scanner, with an in-plane pixel spacing of 0.468 × 0.468 mm, a slice thickness of 0.5 mm, and an inter-slice increment of 0.5 mm (Canon Medical Systems, Otawara, Japan), as verified from the Digital Imaging and Communications in Medicine (DICOM) metadata. In the Korea-recruited cohort, the CBCT images were acquired using a scanner with a voxel size of 0.3 mm (Zenith 3D; Vatech Co., Seoul, Republic of Korea). For the Egypt-recruited cohort, CBCT images were obtained using a scanner with a voxel size of 0.5 mm (Soredex SCANORA 3D; Tuusula, Finland). Craniofacial images were obtained using three different CBCT scanners and one MDCT scanner. CBCT voxel sizes ranged from 0.3 to 0.5 mm. MDCT images had an in-plane pixel spacing of 0.468 × 0.468 mm, a slice thickness of 0.5 mm, and an inter-slice increment of 0.5 mm. Previous studies have examined the comparability of linear measurements across voxel sizes or between CBCT and MDCT [46,47]. These findings do not directly establish comparability for segmentation-derived surfaces, semi-landmark configurations, centroid size, PC scores, or mandibular volume. Imaging modality was included as a covariate in analyses of the full Japan-recruited cohort and in the pooled primary models. A separate CBCT-only sensitivity analysis was also performed in the Japan-recruited cohort. Craniofacial measurements were performed by a single examiner at Kanagawa Dental University using CBCT and MDCT images. Conventional linear, ratio, angular, and volumetric measurements were obtained using Invivo6 (Anatomage, San Jose, CA, USA) and Viewbox4 software (version 4.1.4.1, 64-bit; dHAL Software, Kifissia, Greece) (Figure 1 and Figure 2). Landmark locations and conventional measurement definitions are shown in Figure 1 and Figure 2. The midpoint between the right and left gonion landmarks was defined as the mid-Go, and the midpoint between the right and left condylion landmarks was defined as the mid-Co. The measurement variables were classified as linear, ratio, angular, and volumetric. Linear measurements included sella–nasion (SN) length, ramus length (Co–Go), mandibular body length (Go–Gn), anterior nasal spine–posterior nasal spine (ANS–PNS), intergonial width (Go–Go), intercondylar width (Co–Co), mid-Go-B, mid-Co-B, mid-Co-Gn, mid-Go-Gn, and mid-Co-mid-Go. The ratio variables included ramus length/SN length, mandibular body length/SN length, ramus length/mandibular body length, ramus length/ANS–PNS, mid-Co-Gn/ANS–PNS, mandibular body length/ANS–PNS, SN length/Co–Co, ramus length/Co–Co, mandibular body length/Co–Co, ANS–PNS/Co–Co, Go-Go/Co-Co, mid-Co-mid-Go/Co-Co, mid-Go–Gn/Co–Co, mid-Co–B/Co–Co, mid-Go–B/Co–Co, mid-Co–Gn/Co–Co. The angular measurements included sella–nasion–A point (SNA) angle, sella–nasion–B point (SNB) angle, A point–nasion–B point (ANB) angle, Frankfort-mandibular plane angle (FMA), SN–pogonion (Pog) angle, the difference between the SN–Pog and SNB angles (SN–Pog–SNB difference), the gonial angle (mid-Co–mid-Go–Gn), ∠mid-Go–Gn–B, ∠mid-Go–Pog–B, ∠mid-Go–Pog–Gn, and ∠GoR–Gn–GoL. The ratio variables were calculated to adjust for inter-individual differences in the overall craniofacial size, anteroposterior dimension of the maxilla, and transverse size of the mandible, and to evaluate the relative morphological characteristics of individual craniofacial and mandibular components. SN length, ANS–PNS, and Co–Co were used as representative indices of cranial base length, maxillary length, and transverse mandibular width, respectively. SN length is widely used in cephalometric analysis as a representative measurement of cranial base length and craniofacial size [48]. As the SN length can be associated with mandibular size [49], it was selected as the normalization index for mandibular morphological measurements. The ANS–PNS is commonly used in cephalometric analysis as a representative linear measurement of the anteroposterior dimension of the maxilla or the maxillary length. Therefore, the ANS-PNS was used as a reference measurement to evaluate the relative characteristics of mandibular measurements in relation to the anteroposterior dimension of the maxilla [50]. Co–Co represents the distance between the right and left condylar landmarks, corresponding to the intercondylar width. Because this measurement has been used in previous three-dimensional studies to evaluate the transverse mandibular dimension, Co–Co was selected as the normalization index representing the transverse size of the mandible [51].

The centroid size calculated from the generalized Procrustes analysis (GPA) was used to assess the overall size of the mandible.

Mandibular volume was measured separately using Invivo6 (Anatomage, San Jose, CA, USA), following previously reported CT-based methods [48,52]. In volume-rendering mode, the CT data were trimmed to isolate the mandible, and the dentition was excluded because dental structures can substantially affect volume estimates [52]. Mandibular volume was then calculated using the software’s automatic volume-measurement function and expressed in cubic millimetres (mm^3^).

For geometric morphometric analysis, DICOM data were imported into Mandibular Motion Tracking System (ManMoS, MacroSystem Co., Tokyo, Japan) and 3D Slicer version 5.4.0 for mandibular segmentation and triangular mesh generation. Dentition during mandibular segmentation was excluded because the present study focused exclusively on the bony morphology of the mandible [52], and all the fixed and semi-landmarks were placed on the mandibular bone surface. Three-dimensional landmark and semi-landmark coordinates were obtained by digitizing the mandibular meshes in Viewbox4 using a common template (Figure 3). The template was designed to cover the mandibular condyle, ramus, body, inferior border, and chin region. A total of 354 points were initially digitized, comprising fixed landmarks, curve semi-landmarks, surface semi-landmarks, and one Gnathion helper point. The helper point facilitated semi-landmark placement but was excluded from centroid size calculation, GPA, PCA, and Procrustes ANOVA(R version 4.5.1; geomorph version 4.1.1; RRPP version 2.2.0), leaving 353 analytical landmarks and semi-landmarks.

Only the mandibular mesh used to construct the common template was bilaterally symmetric. The template was used to initialize consistent bilateral landmark and semi-landmark placement across specimens. No symmetrisation or left–right averaging was applied to the individual mandibular meshes or their coordinate configurations. Both sides were retained throughout semi-landmark sliding and subsequent shape analyses, preserving biological asymmetry in the analytical data.

Surface semi-landmarks were placed on the mandibular ramus and around the mandibular condyle. Curves were defined between corresponding fixed landmarks using cubic spline interpolation [53]. The template was transferred to each specimen using thin-plate spline (TPS) deformation and then projected onto the mesh surface using the closest point method. Curve and surface semi-landmarks were slid along their respective curves and mesh surfaces to minimize TPS bending energy and improve correspondence among specimens [54,55]. Landmark and semi-landmark placement, template transfer, surface projection, and mesh-constrained bending-energy sliding were performed through the Viewbox4 graphical user interface; this workflow did not generate an executable user script.

To assess intra-observer repeatability, 30 participants were randomly selected, and their mandibular meshes were redigitized by the same examiner at least two weeks after the initial digitization under the same conditions. The same fixed-landmark placement, semi-landmark placement, and semi-landmark-sliding procedures were applied to both digitizations. Landmark-placement error was quantified using Dahlberg’s formula [56].

### 4.4. Statistical Analysis

To evaluate the overall shape variation in three-dimensional mandibular morphology, GPA was performed using the 353 analytical fixed landmarks and semi-landmarks after exclusion of the Gnathion helper point. GPA standardized the position, rotation, and scale of each specimen. The centroid size calculated during GPA was used as an index of the overall size of the mandible. Centroid size was calculated from the 353 analytical three-dimensional landmark coordinates as the square root of the summed squared distances of all analytical landmarks and semi-landmarks from their centroid. Because the three-dimensional landmark coordinates were recorded in millimetres, raw centroid size was expressed in millimetres. For the association analyses, centroid size was natural-log transformed, and no Z-score standardization was performed. Accordingly, the regression coefficients for this outcome are expressed on the natural-log scale and can be converted to the estimated percentage difference in centroid size per additional derived allele as 100 × (exp(B) − 1). PCA was performed on the Procrustes-aligned shape coordinates, and PC scores were calculated for each participant. Although GPA standardizes uniform scale, it does not by itself remove allometric covariation between shape and centroid size. Natural-log-transformed centroid size was analyzed as a separate secondary outcome and was not included as a covariate in either the primary whole-shape models or the secondary regression models for PC scores and conventional mandibular measurements. Accordingly, these analyses were not designed to estimate genetic effects adjusted for overall mandibular size, and residual allometric covariation may remain in the shape-related results [41]. Starting from the 353-point post-sliding coordinates exported from Viewbox4, centroid size, GPA, and PCA were independently recalculated in R. Because the direction of each PCA axis is arbitrary, the signs of the R-derived PC axes were oriented to correspond to the Viewbox4 axes solely for validation. Before the association analyses, the Viewbox4-derived variables were independently reproduced in R, and the R-derived natural-log-transformed centroid size and PC1–PC11 scores were used in all subsequent statistical analyses. Conventional linear, ratio, angular, and volumetric variables were retained from the original craniofacial measurements. Cohort-specific estimates for each outcome were subsequently combined by meta-analysis. A single PCA was performed using the full 448-participant sample, and the same PC axes were used for all cohort-specific analyses. The whole-shape Procrustes ANOVA was added during revision and was not prespecified in the original analytical framework. In the revised manuscript, it was designated as the primary multivariate analysis of mandibular shape. As the primary multivariate analysis of mandibular shape, whole-shape Procrustes ANOVA was performed in R version 4.5.1 using the generalized Procrustes-aligned coordinates of all 353 analytical landmarks and semi-landmarks, with the geomorph package version 4.1.1 and the RRPP package version 2.2.0 [57]. Separate pooled models were fitted for rs724448 and rs2242116 and included recruitment cohort, sex, mean-centered age, imaging modality, and additive genotype dosage as explanatory variables. Statistical significance was evaluated using residual randomization in a permutation procedure with 9999 random iterations and Type III marginal sums of squares. Bonferroni correction was applied across the two primary SNP tests.

Exploratory whole-shape models were also fitted separately within each cohort, adjusting for sex and age and additionally for imaging modality in the Japan-recruited cohort. The Japan CBCT-only whole-shape sensitivity models adjusted for sex and age. Between-cohort differences in whole-shape genotype associations were evaluated by adding a genotype-by-cohort interaction term to the pooled model containing cohort, sex, age, imaging modality, and genotype.

PC1–PC11 collectively accounted for 70.3% of total shape variance and were retained for secondary descriptive and exploratory analyses. The cumulative-variance percentage was not used to define the primary inferential test. To evaluate the repeatability of the derived geometric morphometric outcomes, the configurations obtained from the repeated digitizations were projected onto the PC axes derived from the original 448-participant analysis. This ensured that the PC scores from the initial and repeated digitizations represented the same dimensions of mandibular shape variation. The intra-observer repeatability of natural-log-transformed centroid size and PC1–PC11 scores was evaluated using a two-way random-effects, absolute-agreement, single-measure ICC(A,1), with 95% confidence intervals [58].

Whole-shape repeatability was additionally assessed using the paired digitizations of the 30 participants. After exclusion of the Gnathion helper point, all 60 configurations of 353 landmarks and semi-landmarks were jointly aligned using GPA. A one-way Procrustes ANOVA partitioned the summed squared variation in the aligned coordinates into between-participant and within-participant components. Whole-shape repeatability was calculated as R, corresponding to two digitizations per participant. This multivariate ICC analogue was calculated separately from the outcome-specific ICC(A,1) estimates. A 95% percentile bootstrap confidence interval was obtained from 5000 resamples of participants, retaining each participant’s paired digitizations and holding the fitted GPA alignment fixed.

To evaluate the association between *PTH1R* polymorphisms and mandibular morphology, multiple linear regression analyses were performed using PC scores as the outcome variables and genotype as the explanatory variable. Additional multiple linear regression analyses were conducted using ln(centroid size), linear measurements, ratio variables, angular measurements, and volumetric measurements as outcome variables to facilitate the interpretation of morphological changes.

Genotypes were analyzed using an additive genetic model and coded as 0, 1, or 2 according to the number of derived alleles. Secondary univariate association analyses were conducted separately within each nationality- and recruitment-defined cohort, with adjustment for sex and age. In the Japan-recruited cohort, imaging modality was additionally included as a covariate because both CBCT and MDCT images were used, with CBCT coded as 0 and MDCT as 1. Stature was not included as a covariate in the analyses of ln(centroid size) in any cohort.

As a sensitivity analysis, the association analyses were repeated after excluding participants examined using MDCT, thereby restricting the analysis to the CBCT-only subgroup. Differences in genotype distributions between the CBCT and MDCT groups were evaluated using Fisher’s exact test.

For the secondary univariate outcomes, cohort-specific regression coefficients and standard errors were combined using an inverse-variance-weighted fixed-effect meta-analysis as the primary summary method [59]. These summary estimates are reported in Table 4 and Table S2. Between-cohort heterogeneity was evaluated for each SNP–outcome combination using Cochran’s Q statistic and quantified using I^2^ [35]. Because three cohorts were included, each Q test had 2 degrees of freedom. With only three cohorts, however, I^2^ may be imprecise or biased and was therefore interpreted cautiously [36]. DerSimonian–Laird random-effects estimates, incorporating the estimated between-cohort variance (τ^2^), were additionally calculated as sensitivity analyses to assess the dependence of the summary results on the choice of meta-analytic model [37]. Differences in statistical significance among the separate cohort analyses were not considered evidence of cohort-specific effects unless supported by formal evidence of between-cohort heterogeneity. Cochran’s Q, heterogeneity *p* values, I^2^ values, and random-effects estimates are reported in Supplementary Table S5; fixed-effect estimates are reported in Table 4 and Table S2.

The primary multivariate shape analysis tested the association of each of the two SNPs with the complete Procrustes shape coordinates. Bonferroni correction was therefore applied across the two primary SNP tests, giving a significance threshold of 0.025. Analyses of natural-log-transformed centroid size, PC1–PC11 scores, and 39 conventional craniofacial measurements were treated as secondary exploratory analyses. For the secondary univariate analyses, Bonferroni and BH-FDR adjustments were applied across 408 tests, comprising 51 outcomes, two SNPs, and four analysis sets: the three cohort-specific analyses and the fixed-effect meta-analysis. These tests were not independent: the meta-analysis incorporated the same cohort-specific estimates, and the two SNPs yielded identical tests in the Korea-recruited cohort because their genotype dosages were identical. The 408-test family was retained to encompass all reported secondary association tests, including these dependent and duplicated tests. Raw and multiple-testing-adjusted *p* values for all 408 secondary tests are provided in Table S7. The 102 between-cohort heterogeneity tests were treated as a separate family and adjusted using both procedures [60,61].

To assess the sensitivity of the rs2242116 results to exclusion of participants with unresolved rs724448 genotype calls, the 51 secondary univariate association analyses were repeated in all 458 eligible participants with available rs2242116 genotypes (Japan, *n* = 200; Korea, *n* = 83; Egypt, *n* = 175). The same additive genetic model and covariate adjustments were used, and cohort-specific estimates were combined by inverse-variance fixed-effect meta-analysis. GPA and PCA were recomputed separately for the original and expanded samples using the 353 analytical points, with PC-axis signs aligned using shared participants. Bonferroni and BH-FDR corrections were applied across a separate family of 204 sensitivity tests, comprising 51 outcomes and four analysis sets: the three cohorts and the fixed-effect meta-analysis. Fixed-effect meta-analysis estimates for the original and expanded samples are compared in Table S8.

An approximate sensitivity analysis was performed to characterize the effect sizes detectable with the available sample sizes. We assumed 80% power, α = 0.025, and a one-degree-of-freedom test of an additive SNP effect in a fixed-effects linear regression model. The pooled model included five covariate degrees of freedom (two for recruitment cohort and one each for sex, age, and imaging modality); the Japan model included three, and the Korea and Egypt models each included two. The corresponding residual degrees of freedom were 441, 194, 75, and 166, respectively. Detectable Cohen’s f^2^ values were calculated using the noncentral F distribution with noncentrality parameter λ = Nf^2^ and converted to partial R^2^ as f^2^/(1 + f^2^). The same α was used across sample sizes for comparison. Calculations were performed in R using the functions qf, pf, and uniroot. This analysis provides a univariate regression benchmark rather than an exact power estimate for the multivariate permutation-based whole-shape analysis.

Statistical analyses were performed using R version 4.5.1 (R Foundation for Statistical Computing, Vienna, Austria) and the Statistical Package for the Social Sciences (SPSS version 28.0; IBM Corporation, Armonk, NY, USA). All tests were two-sided. Unadjusted *p* < 0.05 was described as nominal significance, whereas statistically robust evidence required a multiple-testing-adjusted *p* < 0.05.

### 4.5. GTEx Functional Annotation

During revision, eQTL annotations were verified against GTEx Analysis Release V10 using the GTEx Portal application programming interface (API) (accessed on 30 September 2026). Queries used the Genome Reference Consortium human build 38 (GRCh38) variant identifiers chr3_46894244_A_C_b38 for rs724448 and chr3_46899626_A_G_b38 for rs2242116. Significant single-tissue cis-eQTL records were retrieved without restricting tissue type and filtered for *PTH1R* (ENSG00000160801.14). All six retrieved *PTH1R* variant–tissue associations are presented in Table 5. GTEx reports normalized effect sizes (NES) for the ALT allele relative to the REF allele. Because A was the REF allele for both variants and the effect allele in the mandibular association analyses, the signs of the GTEx NES were reversed to express the effects of allele A relative to the ALT allele; *p* values were unchanged.

## Figures and Tables

**Table 1:** Clinical and imaging characteristics and sagittal skeletal classifications by cohort.

Cohort	*N*	Age (Mean ± SD)	Sex (Male, Female) *n* (%)	Imaging Modality *n*	Class I, *n* (%)	Class II, *n* (%)	Class III, *n* (%)	SNA (Mean ± SD) (°)	SNB (Mean ± SD) (°)	ANB (Mean ± SD) (°)	FMA (Mean ± SD) (°)
Japan	199	28.33 ± 9.79	53 (26.6), 146 (73.4)	CBCT, 158; MDCT, 41	78 (39.2)	89 (44.7)	32 (16.1)	81.00 ± 3.67	77.72 ± 4.81	3.28 ± 3.70	28.29 ± 6.67
Korea	79	25.24 ± 6.51	33 (41.8), 46 (58.2)	CBCT, 79	25 (31.6)	25 (31.6)	29 (36.7)	80.86 ± 3.20	79.81 ± 6.16	1.05 ± 5.28	27.11 ± 7.00
Egypt	170	24.40 ± 5.55	77 (45.3), 93 (54.7)	CBCT, 170	80 (47.1)	80 (47.1)	10 (5.9)	82.45 ± 4.05	78.57 ± 3.96	3.88 ± 2.66	22.32 ± 5.35
Overall	448	26.29 ± 8.06	163 (36.4), 285 (63.6)	CBCT, 407; MDCT, 41	183 (40.8)	194 (43.3)	71 (15.8)	81.52 ± 3.81	78.41 ± 4.84	3.12 ± 3.82	25.82 ± 6.84

Sagittal skeletal patterns were classified according to the ANB angle: Class I, 0° ≤ ANB ≤ 4°; Class II, ANB > 4°; and Class III, ANB < 0°. CT images were originally acquired for clinical orthodontic diagnosis and treatment planning; no additional CT imaging was performed specifically for this study. CBCT, cone-beam computed tomography; MDCT, multidetector computed tomography; SD, standard deviation.

**Table 2:** Allele frequencies and LD coefficients of *PTH1R* SNPs.

rs Number	Chr: Position	Alleles		Derived Allele Frequency			LD Coefficients (Dʹ/r-Squared)		
		Ancestral	Derived	Japan	Korea	Egypt	Japan	Korea	Egypt
rs724448	chr3:46894244	C	A	0.450	0.494	0.597	1.0/0.95	1.0/1.0	0.984/0.669
rs2242116	chr3:46899626	G	A	0.437	0.494	0.506			

LD, linkage disequilibrium; PTH1R, parathyroid hormone 1 receptor; SNP, single-nucleotide polymorphism; Chr, chromosome.

**Table 3:** Whole-shape Procrustes ANOVA of *PTH1R* variants. R^2^ indicates the percentage of total mandibular shape variance explained.

Analysis	Cohort	SNP or Term	*N*	R^2^ (%)	F	*p* Value	Bonferroni *p* Value
A. Primary multivariate genetic analysis							
Pooled analysis of all participants	All	rs724448	448	0.23	1.14	0.28	0.55
		rs2242116	448	0.21	1.08	0.33	0.66
B. Contributions of covariates in the pooled models							
Pooled analysis of all participants	All	Shape differences among cohorts	448	8.52–8.53	21.49–21.52	<0.001	—
		Shape differences by imaging modality	448	1.24–1.25	6.24–6.28	<0.001	—
C. Analysis within each cohort							
Analysis within each cohort	Japan	rs724448	199	0.41	0.85	0.60	—
		rs2242116	199	0.48	1.01	0.41	—
	Korea	rs724448	79	1.41	1.19	0.25	—
		rs2242116	79	1.41	1.19	0.25	—
	Egypt	rs724448	170	0.56	0.97	0.46	—
		rs2242116	170	0.58	1.01	0.41	—
D. Japan CBCT-only sensitivity analysis							
Japan CBCT-only sensitivity analysis	Japan	rs724448	158	0.58	0.93	0.49	0.98
		rs2242116	158	0.76	1.22	0.22	0.44
E. SNP-by-cohort interaction							
Test of SNP-effect differences among cohorts	All	Difference in rs724448 effect among cohorts	448	0.42	1.05	0.37	0.75
		Difference in rs2242116 effect among cohorts	448	0.45	1.14	0.26	0.53

Permutation *p* values were calculated using RRPP with 9999 iterations, and adjusted *p* values were Bonferroni-corrected for the two SNPs. Primary models adjusted for cohort, sex, age, and imaging modality. The recruitment-cohort term includes variation that cannot be separated from scanner-related differences because scanner systems were specific to cohorts. The imaging-modality term distinguishes CBCT from MDCT and does not isolate differences among individual scanner systems. Cohort-specific and CBCT-only analyses were exploratory and sensitivity analyses, respectively; interaction tests evaluated whether SNP effects differed among cohorts. All analyses used 353 landmarks and semi-landmarks. CBCT, cone-beam computed tomography; RRPP, residual randomization in a permutation procedure; ANOVA, analysis of variance.

**Table 4:** Associations of *PTH1R* SNPs with natural-log-transformed mandibular centroid size and mandibular morphology PC scores assessed by multiple regression analysis.

Outcome Variable	Japan (*n* = 199)				Korea (*n* = 79)				Egypt (*n* = 170)				Fixed-Effect Meta-Analysis			
	B	SE	95% CI	*p*	B	SE	95% CI	*p*	B	SE	95% CI	*p*	B	SE	95% CI	*p*
rs724448																
ln(centroid size)	−0.0038	0.0033	−0.0104 to 0.0027	0.25	−0.0020	0.0067	−0.0153 to 0.0113	0.76	−0.0084	0.0038	−0.0159 to −0.0009	0.029 *	−0.0053	0.0023	−0.0099 to −0.0007	0.023 *
PC1	−0.0020	0.0023	−0.0065 to 0.0025	0.38	−0.0039	0.0049	−0.0137 to 0.0059	0.43	−0.0019	0.0027	−0.0072 to 0.0034	0.48	−0.0022	0.0016	−0.0054 to 0.0010	0.19
PC2	0.0018	0.0021	−0.0024 to 0.0059	0.40	0.00055	0.0036	−0.0066 to 0.0077	0.88	0.0024	0.0023	−0.0022 to 0.0069	0.30	0.0018	0.0014	−0.0010 to 0.0046	0.20
PC3	0.00031	0.0017	−0.0031 to 0.0037	0.86	−0.0074	0.0026	−0.0125 to −0.0024	0.0047 *	0.0029	0.0019	−0.0008 to 0.0067	0.12	−0.00030	0.0011	−0.0026 to 0.0020	0.79
PC4	0.00017	0.0013	−0.0025 to 0.0028	0.90	0.000087	0.0022	−0.0043 to 0.0045	0.97	0.00084	0.0017	−0.0025 to 0.0042	0.62	0.00036	0.00095	−0.0015 to 0.0022	0.70
PC5	−0.0023	0.0012	−0.0046 to 0.0000	0.053	0.000044	0.0025	−0.0048 to 0.0049	0.99	0.0016	0.0014	−0.0012 to 0.0044	0.26	−0.00062	0.00085	−0.0023 to 0.0010	0.46
PC6	−0.00026	0.0012	−0.0026 to 0.0021	0.83	−0.00025	0.0023	−0.0048 to 0.0043	0.91	0.00096	0.0013	−0.0016 to 0.0035	0.46	0.00023	0.00081	−0.0014 to 0.0018	0.78
PC7	0.0017	0.0011	−0.0004 to 0.0038	0.10	0.0020	0.0020	−0.0020 to 0.0061	0.32	−0.0014	0.0013	−0.0039 to 0.0011	0.28	0.00068	0.00076	−0.0008 to 0.0022	0.37
PC8	−0.00022	0.00083	−0.0019 to 0.0014	0.79	−0.00041	0.0019	−0.0042 to 0.0034	0.83	0.0028	0.0011	0.0006 to 0.0049	0.013 *	0.00074	0.00063	−0.0005 to 0.0020	0.24
PC9	−0.00045	0.00093	−0.0023 to 0.0014	0.63	0.0041	0.0015	0.0012 to 0.0070	0.0065 *	−0.00077	0.0012	−0.0032 to 0.0017	0.53	0.00039	0.00066	−0.0009 to 0.0017	0.56
PC10	0.00065	0.00079	−0.0009 to 0.0022	0.41	0.0013	0.0015	−0.0017 to 0.0043	0.39	0.00072	0.0011	−0.0015 to 0.0029	0.52	0.00077	0.00059	−0.0004 to 0.0019	0.19
PC11	0.00045	0.00078	−0.0011 to 0.0020	0.56	0.0014	0.0012	−0.0010 to 0.0038	0.25	0.00056	0.00098	−0.0014 to 0.0025	0.57	0.00068	0.00054	−0.0004 to 0.0017	0.21
rs2242116																
ln(centroid size)	−0.0048	0.0033	−0.0113 to 0.0017	0.14	−0.0020	0.0067	−0.0153 to 0.0113	0.76	−0.0073	0.0037	−0.0147 to 0.0001	0.052	−0.0054	0.0023	−0.0100 to −0.0009	0.019 *
PC1	−0.0017	0.0023	−0.0061 to 0.0028	0.47	−0.0039	0.0049	−0.0137 to 0.0059	0.43	0.0014	0.0026	−0.0037 to 0.0066	0.58	−0.00071	0.0016	−0.0039 to 0.0025	0.66
PC2	0.0028	0.0021	−0.0013 to 0.0069	0.18	0.00055	0.0036	−0.0066 to 0.0077	0.88	0.0024	0.0022	−0.0020 to 0.0068	0.27	0.0023	0.0014	−0.0004 to 0.0051	0.10
PC3	0.000062	0.0017	−0.0033 to 0.0035	0.97	−0.0074	0.0026	−0.0125 to −0.0024	0.0047 *	0.0020	0.0019	−0.0017 to 0.0057	0.29	−0.00071	0.0011	−0.0029 to 0.0015	0.53
PC4	0.00068	0.0013	−0.0020 to 0.0033	0.61	0.000087	0.0022	−0.0043 to 0.0045	0.97	0.0015	0.0017	−0.0017 to 0.0048	0.36	0.00085	0.00094	−0.0010 to 0.0027	0.37
PC5	−0.0025	0.0012	−0.0048 to −0.0002	0.031 *	0.000044	0.0025	−0.0048 to 0.0049	0.99	0.00017	0.0014	−0.0026 to 0.0029	0.90	−0.0013	0.00084	−0.0029 to 0.0004	0.13
PC6	−0.00042	0.0012	−0.0027 to 0.0019	0.72	−0.00025	0.0023	−0.0048 to 0.0043	0.91	0.0016	0.0013	−0.0009 to 0.0041	0.21	0.00042	0.00080	−0.0011 to 0.0020	0.60
PC7	0.0016	0.0011	−0.0005 to 0.0037	0.13	0.0020	0.0020	−0.0020 to 0.0061	0.32	−0.0012	0.0013	−0.0036 to 0.0013	0.36	0.00067	0.00075	−0.0008 to 0.0021	0.37
PC8	−0.00017	0.00083	−0.0018 to 0.0015	0.84	−0.00041	0.0019	−0.0042 to 0.0034	0.83	0.0029	0.0011	0.0008 to 0.0050	0.0079 *	0.00083	0.00062	−0.0004 to 0.0020	0.18
PC9	−0.00065	0.00092	−0.0025 to 0.0012	0.48	0.0041	0.0015	0.0012 to 0.0070	0.0065 *	−0.00040	0.0012	−0.0028 to 0.0020	0.74	0.00037	0.00065	−0.0009 to 0.0017	0.57
PC10	0.00046	0.00078	−0.0011 to 0.0020	0.56	0.0013	0.0015	−0.0017 to 0.0043	0.39	−0.00056	0.0011	−0.0027 to 0.0016	0.60	0.00029	0.00058	−0.0009 to 0.0014	0.62
PC11	0.00049	0.00077	−0.0010 to 0.0020	0.53	0.0014	0.0012	−0.0010 to 0.0038	0.25	0.00055	0.00096	−0.0013 to 0.0024	0.57	0.00069	0.00054	−0.0004 to 0.0017	0.20

* indicates a nominal association (*p* < 0.05). The number of derived alleles (0–2) was used as the explanatory variable. Sex and age were included as covariates in all cohort-specific analyses, with imaging modality included as an additional covariate in the Japan-recruited cohort. Fixed-effect meta-analysis estimates were calculated by combining the cohort-specific regression coefficients and standard errors using inverse-variance weighting. In the Korea-recruited cohort, rs724448 and rs2242116 were in complete linkage disequilibrium (r^2^ = 1.0); therefore, their identical regression estimates reflect redundant genotype information and should not be interpreted as independent association signals. PC, principal component; PTH1R, parathyroid hormone 1 receptor; SNP, single-nucleotide polymorphism; B, regression coefficient; SE, standard error; CI, confidence interval.

**Table 5:** GTEx eQTL associations of *PTH1R* SNPs rs724448 and rs2242116 with *PTH1R* expression in adipose and breast tissues.

SNP	Variant ID	ALT Allele	REF Allele	Effect Allele	Gene	Tissue	*p* Value	NES (Effect Allele A)
rs724448	chr3_46894244_A_C_b38	C	A	A	*PTH1R*	Adipose-Visceral (Omentum)	2.3 × 10^−7^	+0.15
						Adipose-Subcutaneous	2.0 × 10^−6^	+0.13
						Breast-Mammary Tissue	6.5 × 10^−5^	+0.10
rs2242116	chr3_46899626_A_G_b38	G	A	A		Adipose-Visceral (Omentum)	6.3 × 10^−7^	+0.14
						Breast-Mammary Tissue	7.7 × 10^−5^	+0.09
						Adipose-Subcutaneous	1.0 × 10^−4^	+0.10

Data were obtained from the GTEx Portal, GTEx Analysis Release V10 (https://gtexportal.org/home/), and verified against the Portal API on 30 September 2026. NES values are expressed for the A allele, which was the effect allele in the mandibular association analyses and the REF allele for both variants. The signs of the original ALT-based GTEx NES were reversed; *p* values were unchanged. GTEx, Genotype-Tissue Expression; eQTL, expression quantitative trait locus; PTH1R, parathyroid hormone 1 receptor; SNP, single-nucleotide polymorphism; ALT allele, alternative allele; NES, normalized effect sizes; REF allele, reference allele.

## Data Availability

The R analysis code, computational environment information, validation outputs, non-participant-level statistical results, and Viewbox4 template materials are publicly available on Zenodo at https://doi.org/10.5281/zenodo.23094886. Simulated input data, execution instructions, and verified example outputs are also provided to demonstrate execution of the deposited analysis scripts. These data contain no actual participant observations and are not intended to reproduce the study’s numerical results. Landmark and semi-landmark placement, template transfer, surface projection, and mesh-constrained bending-energy sliding were performed using the graphical user interface of Viewbox4. As these preprocessing procedures were carried out within Viewbox4, they are not reproduced by the deposited R code, and no separate executable script was generated for these procedures. The deposited Viewbox4 materials include the 354-point XML template dataset and the corresponding mandibular template mesh. Actual participant-level genotype, phenotype, PCA-score, landmark, and Procrustes-coordinate data are not included in the public repository to safeguard participant privacy and comply with applicable informed-consent, institutional ethics, and privacy requirements. Participant-level data are available under controlled access, subject to applicable consent, ethics, and institutional requirements. Requests should be directed to the corresponding author, Tetsutaro Yamaguchi, for review by the relevant institutions.

## Supplementary Materials

The following supporting information can be downloaded at https://www.mdpi.com/article/10.3390/ijms27198894/s1.

## Abbreviations

The following abbreviations are used in this manuscript:

ALT	Alternative allele
Ihh	Indian hedgehog
CBCT	Cone-beam computed tomography
MDCT	Multidetector computed tomography
CT	Computed tomography
DICOM	Digital Imaging and Communications in Medicine
ANB	A point–nasion–B point
SN	sella–nasion
eQTL	Expression quantitative trait locus
GPA	Generalized Procrustes Analysis
GTEx	Genotype-Tissue Expression
GWAS	Genome-wide association study
JMC	Jansen metaphyseal chondrodysplasia
LD	Linkage disequilibrium
NES	Normalized effect size
PC	Principal component
PCA	Principal component analysis
PFE	Primary failure of eruption
PTH	Parathyroid hormone
PTH1R	Parathyroid hormone 1 receptor
PTHrP	Parathyroid hormone-related protein
REF	Reference allele
SNP	Single-nucleotide polymorphism
TPS	Thin-plate spline
SD	Standard deviation
ICC	Intraclass correlation coefficient
CI	Confidence interval
ANOVA	Analysis of variance
BH-FDR	Benjamini–Hochberg false-discovery rate
GRCh38	Genome Reference Consortium human build 38
API	Application programming interface
