# Supplementary material for: Association of PTH1R Gene Polymorphisms with Three-Dimensional Mandibular Morphology Across Three Recruitment Cohorts
Source: Int J Mol Sci. 2026 Oct 7;27(19):8894. doi: 10.3390/ijms27198894 (PMC13665180; doi:10.3390/ijms27198894)
Supplement: Supplementary file 1 [file ijms-27-08894-s001.zip › ijms-4606966-supplementary.pdf]

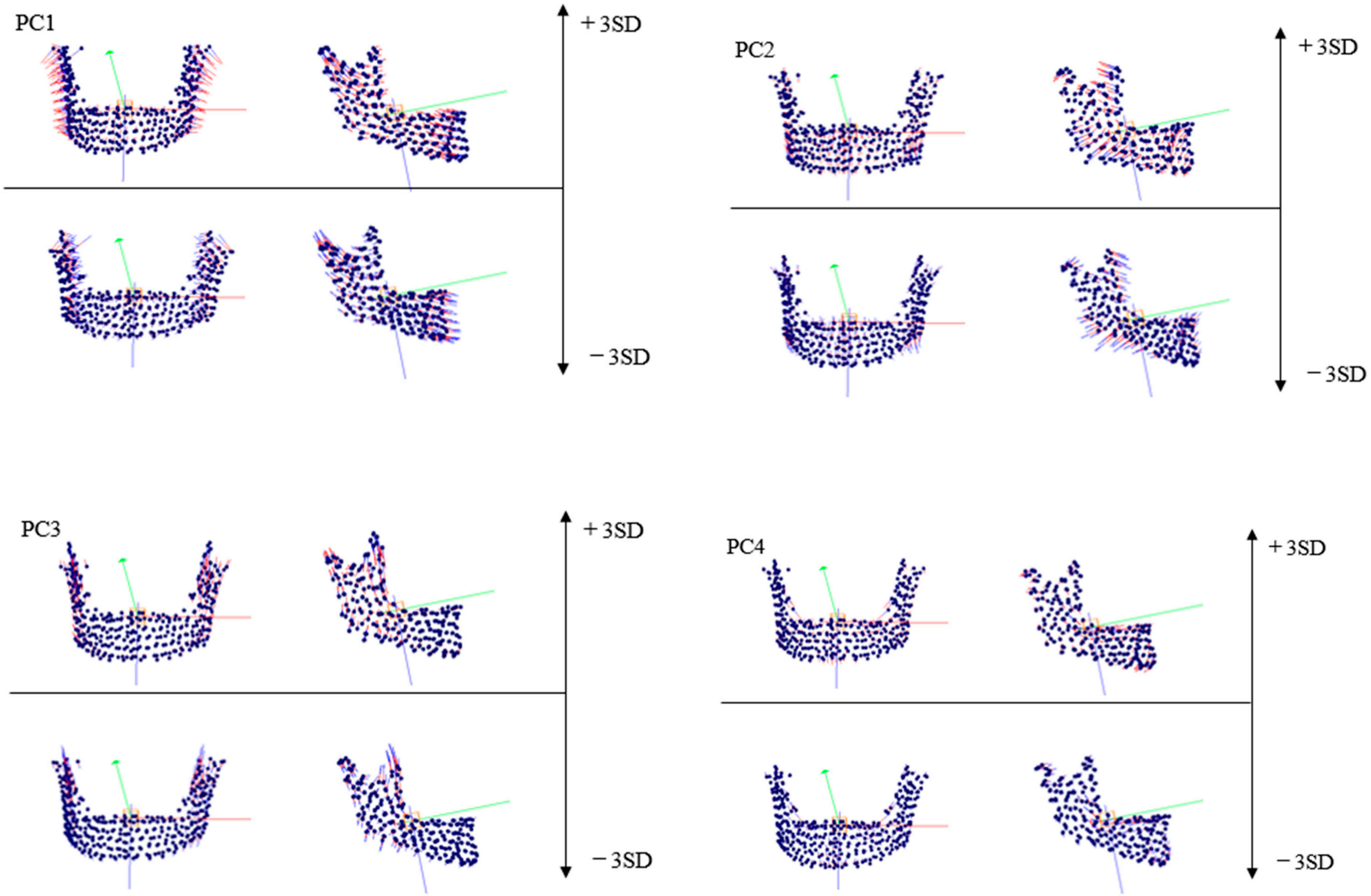
PC1
+3SD
−3SD
PC2
+3SD
−3SD
PC3
+3SD
−3SD
PC4
+3SD
−3SD

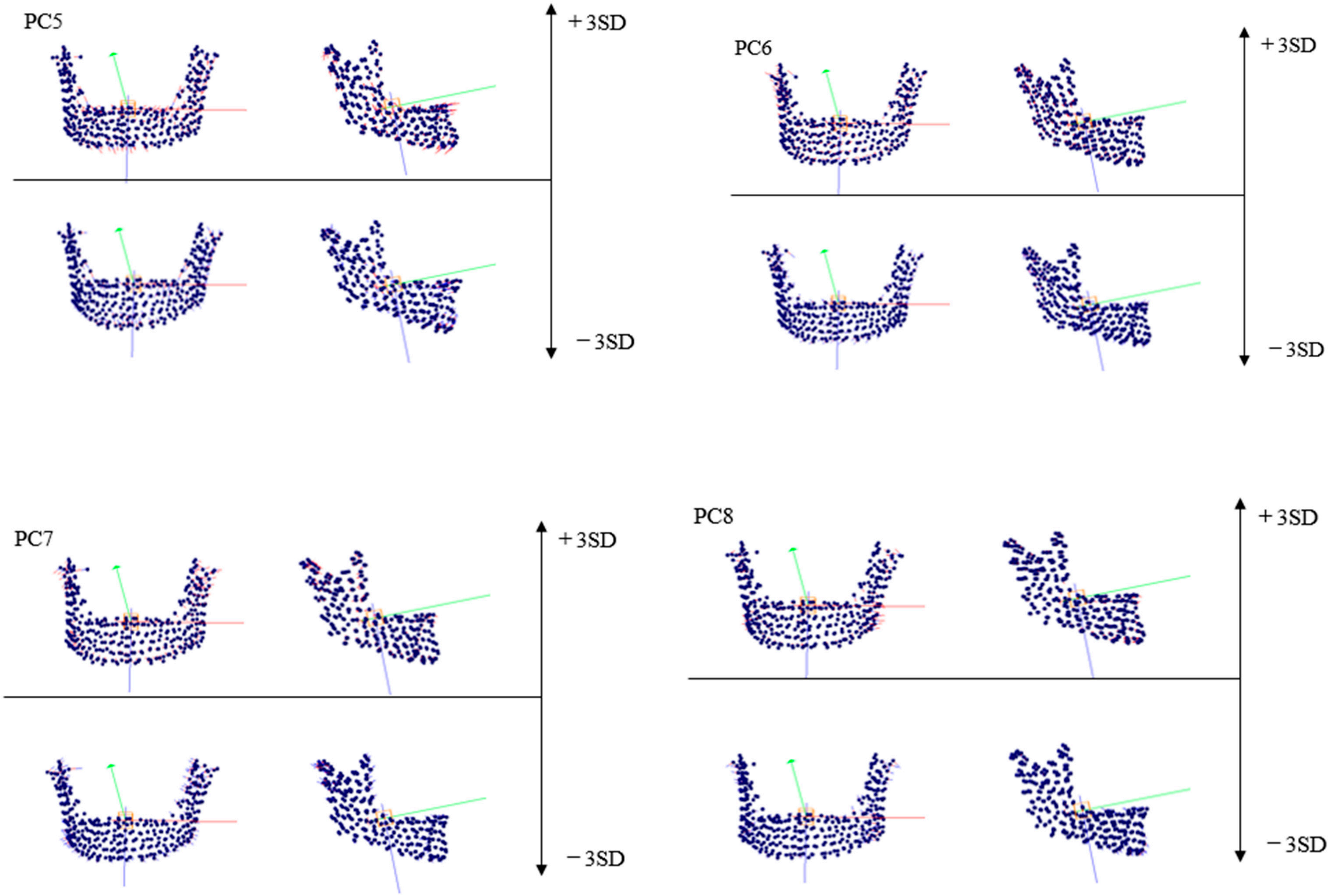
PC5
+3SD
−3SD
PC6
+3SD
−3SD
PC7
+3SD
−3SD
PC8
+3SD
−3SD

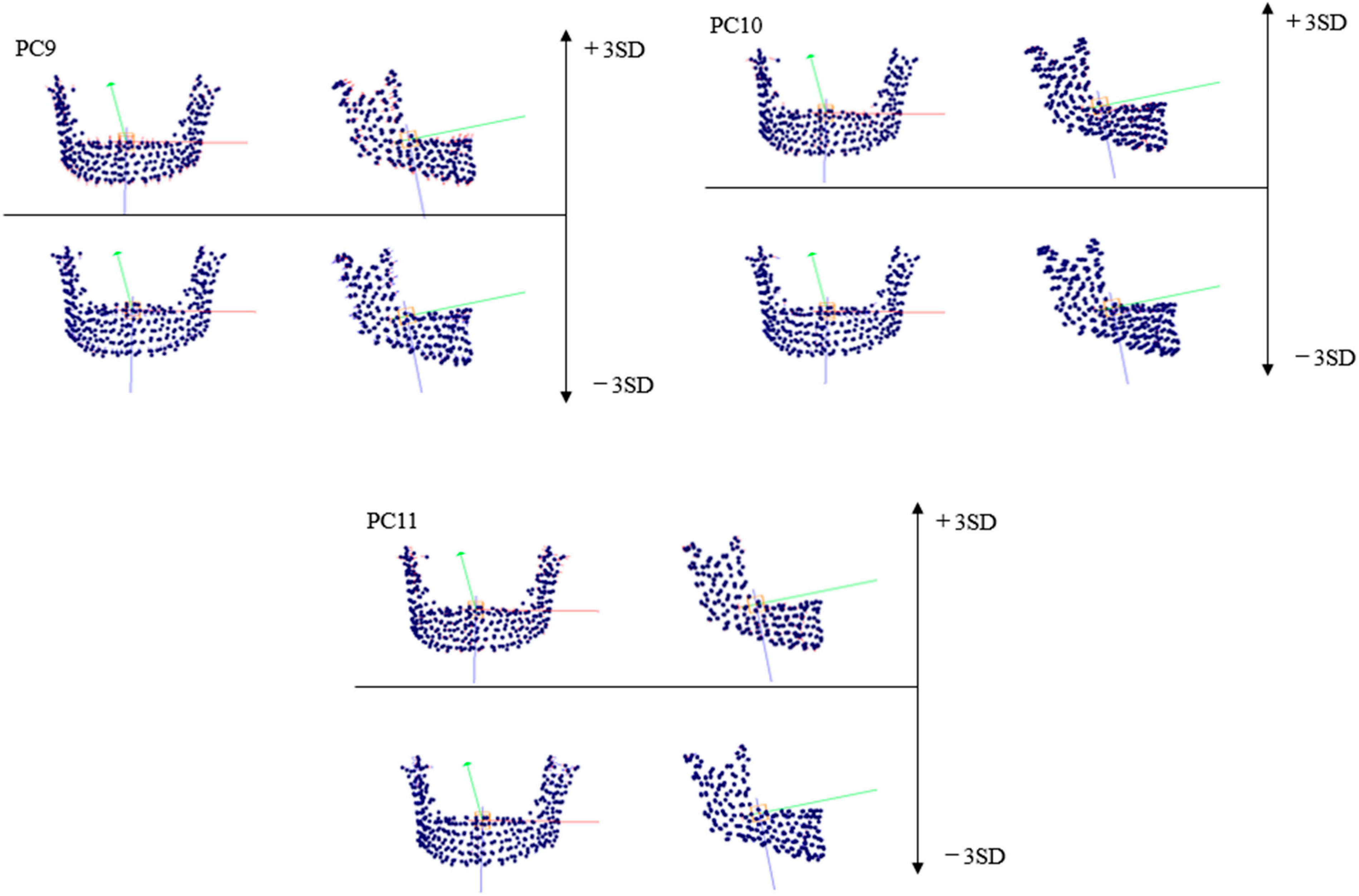
PC9
+3SD
−3SD
PC10
+3SD
−3SD
PC11
+3SD
−3SD

Figure S1. Three-dimensional mandibular shape variation along PC1–PC11. For each principal component, mandibular configurations at +3 SD and −3 SD from the mean are shown in frontal and lateral views. PC, principal component; SD, standard deviation.

Table S1A. Genotyping call rates and repeat testing.

Recruitment cohort	SNP	Eligible n	Initial no-calls, n (%)	Initial call rate %	Re-genotyped	Resolved	Final no-calls	Final called n	Final call rate %
Japan	rs724448	200	4 (2.0%)	98.00%	4	3	1	199	99.50%
	rs2242116		4 (2.0%)	98.00%	4	4	0	200	100.00%
Egypt	rs724448	175	23 (13.1%)	86.90%	23	18	5	170	97.10%
	rs2242116		0 (0.0%)	100.00%	0	0	0	175	100.00%
Korea	rs724448	83	10 (12.0%)	88.00%	10	6	4	79	95.20%
	rs2242116		0 (0.0%)	100.00%	0	0	0	83	100.00%
Overall	rs724448	458	37 (8.1%)	91.90%	37	27	10	448	97.80%
	rs2242116		4 (0.9%)	99.10%	4	4	0	458	100.00%

Eligible n denotes all samples entering genotyping quality control (Japan, n = 200; Korea, n = 83; Egypt, n = 175). Initial no-call denotes a sample for which no genotype was assigned in the first assay. Re-genotyped denotes initial no-call samples subjected to repeat testing; resolved denotes those for which a genotype was assigned after repeat testing; and final no-call denotes samples remaining unresolved after repeat testing. Initial and final call rates were calculated as the proportions of eligible samples with an assigned genotype before and after repeat testing, respectively. Ten samples with a final no-call for rs724448 were excluded, yielding complete genotype data for both SNPs in 448 participants. Twenty samples were genotyped in duplicate, with 100% concordance for both SNPs. SNP, single-nucleotide polymorphism.

Table S1B. Genotype counts, minor allele frequencies, and Hardy-Weinberg equilibrium results by recruitment cohort.

Recruitment cohort	SNP	Analytical n	Genotype counts, n	Minor allele	MAF	HWE exact *p*
Japan	rs724448	199	C/C: 64 A/C: 91 A/A: 44	A	0.45	0.32
	rs2242116		G/G: 68 A/G: 88 A/A: 43	A	0.44	0.15
Egypt	rs724448	170	C/C: 27 A/C: 83 A/A: 60	C	0.40	1.0
	rs2242116		G/G: 41 A/G: 86 A/A: 43	G	0.49	1.0
Korea	rs724448	79	C/C: 20 A/C: 40 A/A: 19	A	0.49	1.0
	rs2242116		G/G: 20 A/G: 40 A/A: 19	A	0.49	1.0

Genotype counts are reported after final genotyping quality control in the complete-case analytical sample. For rs724448, genotypes are shown as C/C, A/C, and A/A; for rs2242116, genotypes are shown as G/G, A/G, and A/A. The derived A allele was used as the effect allele in the association analyses for both SNPs and was coded additively as 0, 1, or 2 copies. MAF denotes the frequency of the less frequent allele within each recruitment cohort and therefore is not necessarily identical to the effect-allele frequency. HWE P values were calculated using an exact test; neither SNP showed a statistically significant deviation from HWE in any cohort (all exact p values ≥ 0.152). MAF, minor allele frequency; HWE, Hardy-Weinberg equilibrium; SNP, single-nucleotide polymorphism.

Figure S2. Allelic discrimination plots for rs724448 showing initial and repeat genotyping of samples with persistently unresolved calls.

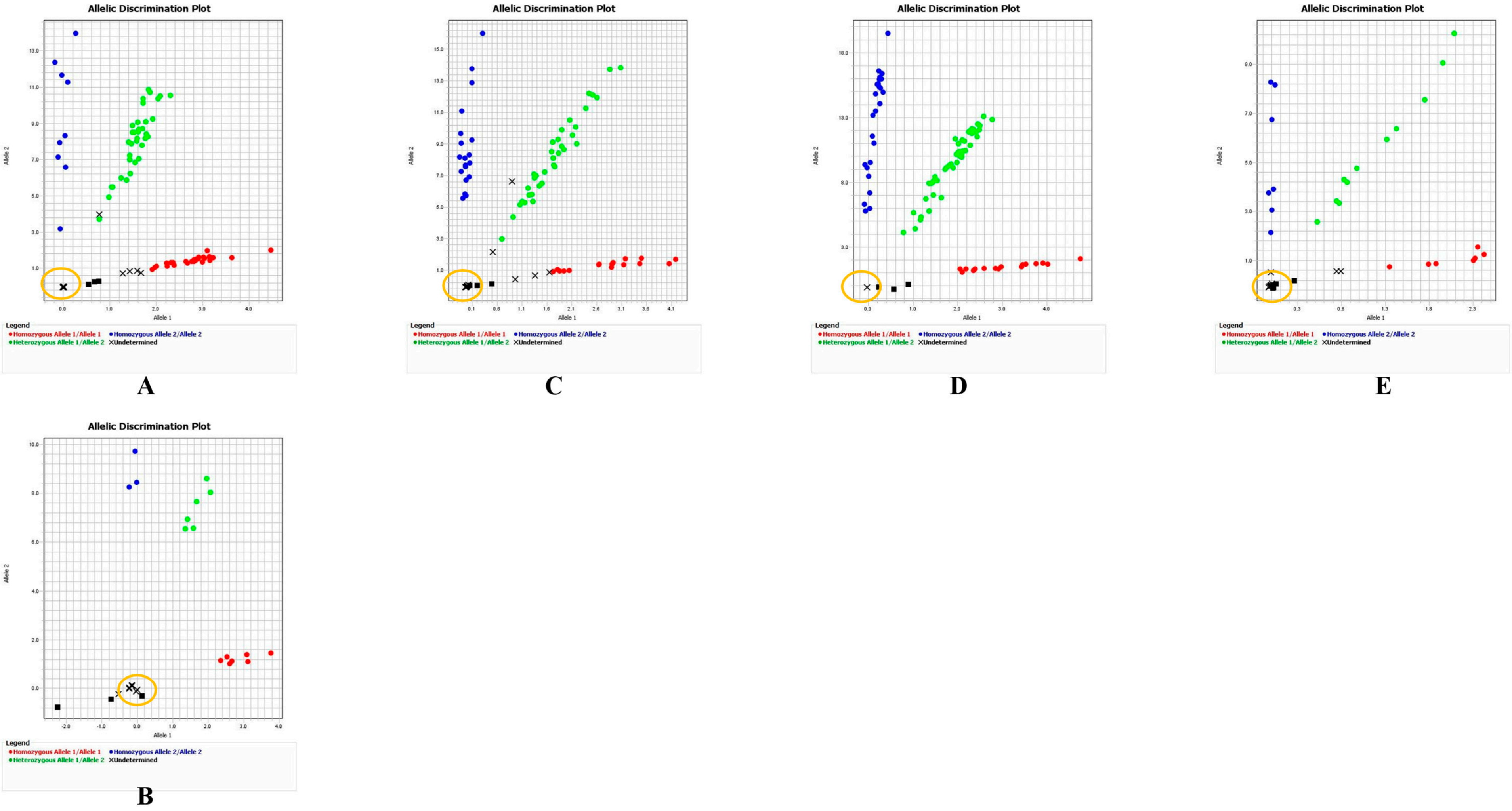

Panels A–D show the initial genotyping runs containing the 10 samples whose rs724448 calls remained unresolved after repeat testing: three samples recruited in Egypt (A), two recruited in Egypt (B), four recruited in Korea (C), and one recruited in Japan (D). Panel E shows the repeat genotyping run containing these same 10 samples. The sample counts indicate the persistently unresolved samples of interest, not the total number of reactions displayed in each panel. Other reactions within the displayed runs are retained to provide context for the genotype clusters. The horizontal and vertical axes represent fluorescence signals for Allele 1 and Allele 2, respectively. Red, blue, and green points represent calls classified as Allele A homozygotes, Allele C homozygotes, and heterozygotes, respectively. Black crosses indicate undetermined reactions, and black squares indicate no-template controls (NTCs). Orange circles highlight regions containing the persistently unresolved samples; NTCs may also lie within these regions. Some plotted points overlap. The highlighted samples showed low fluorescence signals in both allele channels and remained uncalled after repeat genotyping. These plots focus on the 10 final unresolved samples; initial and final no-call counts for the full study sample are reported in Table S1A.

Figure S3. Shape variation along PC8 is visualized as deviations of ±3 SD from the mean three-dimensional coordinates. (**a**) Lateral view. (**b**) Anterior view.
PC8, principal component 8; SD, standard deviation.

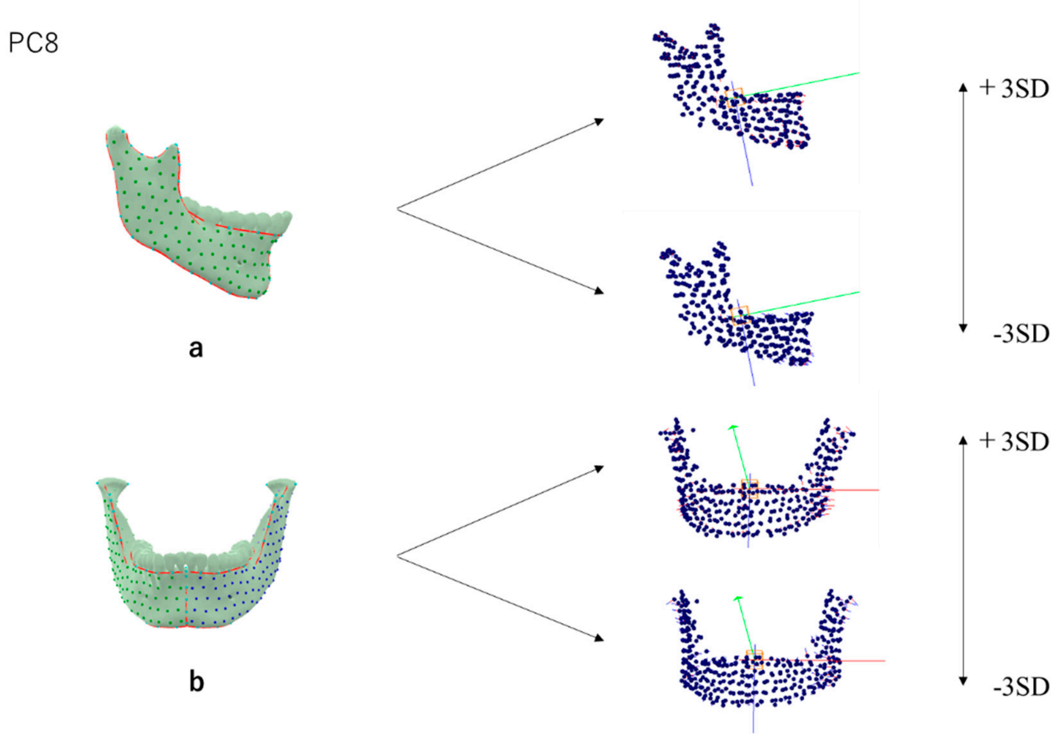

Table S2. Association of PTH1R SNPs with mandibular morphological measurements assessed using single-SNP multiple regression models.

Category	Measurements	Japan (n=199)				Korea (n=79)				Egypt (n=170)				Fixed-effect meta-analysis			
		B	SE	95% CI	*p*-value	B	SE	95% CI	*p*-value	B	SE	95% CI	*p*-value	B	SE	95% CI	*p*-value
	rs724448																
volumetric measurements	Volume	-107	594	-1280 to 1065	0.86	-39	1111	-2252 to 2174	0.97	-396	661	-1701 to 909	0.55	-210	411	-1014 to 595	0.61
linear measurements	SN length	0.12	0.29	-0.44 to 0.69	0.67	-0.19	0.54	-1.28 to 0.89	0.72	-0.78	0.33	-1.43 to -0.12	0.020*	-0.25	0.20	-0.65 to 0.14	0.21
	ramus length	-0.15	0.41	-0.96 to 0.66	0.72	0.062	0.81	-1.55 to 1.67	0.94	-0.28	0.48	-1.22 to 0.66	0.56	-0.17	0.29	-0.74 to 0.40	0.56
	mandibular body length	-0.26	0.39	-1.02 to 0.51	0.51	-0.63	0.88	-2.38 to 1.13	0.48	-1.1	0.43	-1.91 to -0.20	0.016*	-0.61	0.27	-1.15 to -0.08	0.025*
	ANS-PNS	0.17	0.29	-0.41 to 0.75	0.56	0.33	0.62	-0.91 to 1.57	0.59	-1.1	0.42	-1.93 to -0.28	0.0086*	-0.18	0.22	-0.62 to 0.26	0.43
	intergonial width (Go-Go)	-0.23	0.56	-1.33 to 0.88	0.69	1.2	0.88	-0.56 to 2.96	0.18	-1.7	0.67	-3.00 to -0.38	0.012*	-0.45	0.39	-1.20 to 0.31	0.25
	intercondylar width (Co-Co)	-0.22	0.51	-1.23 to 0.78	0.66	0.71	0.99	-1.26 to 2.68	0.48	0.16	0.60	-1.03 to 1.35	0.79	0.041	0.36	-0.67 to 0.75	0.91
	mid-Go-B	-0.12	0.38	-0.88 to 0.64	0.76	-0.42	0.92	-2.24 to 1.41	0.65	-0.76	0.46	-1.67 to 0.16	0.11	-0.38	0.28	-0.93 to 0.17	0.18
	mid-Co–B	-0.22	0.53	-1.26 to 0.83	0.68	-0.22	1.0	-2.30 to 1.86	0.83	-1.3	0.52	-2.32 to -0.26	0.014*	-0.70	0.35	-1.39 to -0.02	0.045*
	mid-Co–Gn	-0.47	0.57	-1.60 to 0.65	0.41	-0.52	1.2	-2.89 to 1.85	0.66	-1.3	0.57	-2.40 to -0.16	0.025*	-0.84	0.38	-1.59 to -0.10	0.027*
	mid-Go–Gn	-0.35	0.42	-1.17 to 0.47	0.40	-0.41	1.0	-2.41 to 1.59	0.68	-0.88	0.48	-1.83 to 0.07	0.069	-0.56	0.30	-1.15 to 0.03	0.061
	mid-Co–mid-Go	0.054	0.44	-0.81 to 0.92	0.90	-0.31	0.95	-2.19 to 1.58	0.75	-0.12	0.47	-1.05 to 0.82	0.81	-0.054	0.30	-0.65 to 0.54	0.86
ratio variables	ramus length/SN length	-0.0042	0.0076	-0.019 to 0.011	0.58	0.0019	0.014	-0.026 to 0.030	0.89	0.0063	0.0074	-0.008 to 0.021	0.40	0.0013	0.0050	-0.008 to 0.011	0.80
	mandibular body length/SN length	-0.0062	0.0075	-0.021 to 0.009	0.42	-0.0073	0.015	-0.036 to 0.022	0.62	-0.00061	0.0079	-0.016 to 0.015	0.94	-0.0040	0.0051	-0.014 to 0.006	0.44
	ramus length/mandibular body length	0.000057	0.0048	-0.009 to 0.010	0.99	0.0049	0.0075	-0.010 to 0.020	0.52	0.0048	0.0058	-0.007 to 0.016	0.41	0.0025	0.0033	-0.004 to 0.009	0.45
	ramus length/ANS-PNS	-0.0085	0.011	-0.030 to 0.013	0.43	-0.0079	0.023	-0.054 to 0.039	0.74	0.020	0.012	-0.004 to 0.044	0.10	0.0026	0.0076	-0.012 to 0.017	0.73
	mid-Co–Gn/ANS–PNS	-0.020	0.018	-0.057 to 0.016	0.27	-0.026	0.039	-0.105 to 0.052	0.51	0.032	0.021	-0.009 to 0.073	0.13	-0.00039	0.013	-0.026 to 0.025	0.98
	mandibular body length/ANS–PNS	-0.012	0.012	-0.036 to 0.012	0.31	-0.027	0.027	-0.082 to 0.028	0.33	0.017	0.014	-0.011 to 0.045	0.23	-0.0027	0.0087	-0.020 to 0.014	0.75
	SN length/Co–Co	0.0024	0.0039	-0.005 to 0.010	0.54	-0.0050	0.0070	-0.019 to 0.009	0.48	-0.0090	0.0053	-0.019 to 0.001	0.089	-0.0022	0.0029	-0.008 to 0.003	0.44
	ramus length/Co–Co	-0.00014	0.0046	-0.009 to 0.009	0.98	-0.0023	0.0093	-0.021 to 0.016	0.81	-0.0037	0.0055	-0.014 to 0.007	0.50	-0.0017	0.0033	-0.008 to 0.005	0.61
	mandibular body length/Co–Co	-0.00055	0.0047	-0.010 to 0.009	0.91	-0.010	0.010	-0.030 to 0.010	0.33	-0.012	0.0062	-0.024 to 0.000	0.051	-0.0054	0.0035	-0.012 to 0.001	0.12
	ANS–PNS/Co–Co	0.0026	0.0038	-0.005 to 0.010	0.49	0.0010	0.0061	-0.011 to 0.013	0.88	-0.012	0.0050	-0.021 to -0.002	0.022*	-0.0019	0.0027	-0.007 to 0.003	0.49
	Go–Go/Co–Co	-0.000091	0.0054	-0.011 to 0.011	0.99	0.0066	0.011	-0.015 to 0.028	0.55	-0.018	0.0070	-0.032 to -0.005	0.010*	-0.0051	0.0040	-0.013 to 0.003	0.20
	mid-Co–mid-Go/Co–Co	0.0017	0.0049	-0.008 to 0.011	0.74	-0.0057	0.011	-0.027 to 0.015	0.59	-0.0020	0.0055	-0.013 to 0.009	0.71	-0.00058	0.0035	-0.007 to 0.006	0.87
	mid-Go–Gn/Co–Co	-0.0017	0.0049	-0.011 to 0.008	0.73	-0.0074	0.011	-0.029 to 0.014	0.49	-0.010	0.0064	-0.023 to 0.002	0.11	-0.0051	0.0037	-0.012 to 0.002	0.16
	mid-Co–B/Co–Co	0.00010	0.0071	-0.014 to 0.014	0.99	-0.0066	0.014	-0.035 to 0.021	0.64	-0.015	0.0082	-0.031 to 0.001	0.069	-0.0064	0.0050	-0.016 to 0.003	0.20

Category	Measurements	Japan (n=199)				Korea (n=79)				Egypt (n=170)				Fixed-effect meta-analysis			
		B	SE	95% CI	*p*-value	B	SE	95% CI	*p*-value	B	SE	95% CI	*p*-value	B	SE	95% CI	*p*-value
	mid-Go–B/Co–Co	0.00058	0.0047	-0.009 to 0.010	0.90	-0.0070	0.010	-0.027 to 0.013	0.48	-0.0088	0.0064	-0.021 to 0.004	0.17	-0.0032	0.0035	-0.010 to 0.004	0.36
	mid-Co–Gn/Co–Co	-0.0022	0.0075	-0.017 to 0.013	0.77	-0.010	0.015	-0.041 to 0.021	0.51	-0.015	0.0086	-0.032 to 0.002	0.087	-0.0079	0.0053	-0.018 to 0.002	0.14
angular measurements	SNA	-0.18	0.36	-0.88 to 0.52	0.62	0.39	0.52	-0.65 to 1.43	0.46	0.21	0.46	-0.69 to 1.12	0.64	0.064	0.25	-0.42 to 0.55	0.80
	SNB	-0.62	0.44	-1.49 to 0.25	0.16	-0.29	0.96	-2.20 to 1.62	0.76	-0.066	0.44	-0.94 to 0.81	0.88	-0.34	0.30	-0.92 to 0.24	0.25
	ANB	0.44	0.33	-0.21 to 1.09	0.18	0.69	0.79	-0.89 to 2.26	0.39	0.28	0.30	-0.31 to 0.87	0.35	0.38	0.21	-0.04 to 0.79	0.077
	FMA	0.025	0.62	-1.20 to 1.25	0.97	-0.10	1.1	-2.31 to 2.11	0.93	-0.51	0.60	-1.69 to 0.68	0.40	-0.23	0.40	-1.02 to 0.56	0.57
	S–N–Pog angle	-0.59	0.45	-1.48 to 0.30	0.20	-0.98	1.0	-3.06 to 1.10	0.35	-0.18	0.45	-1.07 to 0.71	0.69	-0.44	0.31	-1.04 to 0.16	0.15
	S–N–Pog–SNB difference	0.033	0.22	-0.40 to 0.46	0.88	-0.69	0.36	-1.41 to 0.03	0.060	-0.11	0.17	-0.45 to 0.22	0.50	-0.14	0.12	-0.38 to 0.11	0.28
	Gonial angle (mid-Co–mid-Go–Gn)	-0.048	0.51	-1.05 to 0.96	0.93	0.26	0.95	-1.64 to 2.15	0.79	0.0073	0.51	-0.99 to 1.01	0.99	0.014	0.34	-0.64 to 0.67	0.97
	mid-Go–Gn–B	0.49	0.50	-0.49 to 1.47	0.33	-0.15	0.85	-1.84 to 1.54	0.86	0.20	0.55	-0.89 to 1.28	0.72	0.28	0.34	-0.39 to 0.94	0.41
	mid-Go–Pog–B	0.40	0.51	-0.61 to 1.41	0.43	-0.23	0.98	-2.18 to 1.71	0.81	0.26	0.61	-0.94 to 1.45	0.67	0.26	0.36	-0.45 to 0.97	0.47
	mid-Go–Pog–Gn	-0.43	0.73	-1.87 to 1.02	0.56	0.63	1.4	-2.06 to 3.32	0.64	-0.26	0.78	-1.80 to 1.27	0.73	-0.22	0.50	-1.19 to 0.75	0.66
	GoR–Gn–GoL	0.10	0.41	-0.71 to 0.90	0.81	1.0	0.88	-0.75 to 2.75	0.26	-0.34	0.50	-1.33 to 0.65	0.50	0.048	0.30	-0.53 to 0.63	0.87
	rs2242116																
volumetric measurements	Volume	-129	589	-1291 to 1033	0.83	-39	1111	-2252 to 2174	0.97	339	646	-936 to 1613	0.60	67	405	-727 to 861	0.87
linear measurements	SN length	0.053	0.29	-0.51 to 0.62	0.85	-0.19	0.54	-1.28 to 0.89	0.72	-0.61	0.32	-1.25 to 0.03	0.063	-0.23	0.20	-0.62 to 0.16	0.25
	ramus length	-0.079	0.41	-0.89 to 0.73	0.85	0.062	0.81	-1.55 to 1.67	0.94	-0.0011	0.46	-0.92 to 0.92	1.0	-0.031	0.29	-0.59 to 0.53	0.91
	mandibular body length	-0.36	0.38	-1.11 to 0.40	0.35	-0.63	0.88	-2.38 to 1.13	0.48	-0.69	0.43	-1.53 to 0.15	0.11	-0.52	0.27	-1.05 to 0.01	0.056
	ANS-PNS	0.23	0.29	-0.34 to 0.80	0.43	0.33	0.62	-0.91 to 1.57	0.59	-0.80	0.41	-1.61 to 0.01	0.052	-0.059	0.22	-0.49 to 0.38	0.79
	intergonial width (Go-Go)	-0.45	0.56	-1.55 to 0.64	0.42	1.2	0.88	-0.56 to 2.96	0.18	-1.8	0.65	-3.06 to -0.50	0.0067*	-0.60	0.38	-1.35 to 0.14	0.11
	intercondylar width (Co-Co)	-0.32	0.51	-1.32 to 0.68	0.53	0.71	0.99	-1.26 to 2.68	0.48	-0.14	0.59	-1.30 to 1.02	0.81	-0.12	0.36	-0.82 to 0.58	0.74
	mid-Go-B	-0.063	0.38	-0.81 to 0.69	0.87	-0.42	0.92	-2.24 to 1.41	0.65	-0.20	0.46	-1.10 to 0.71	0.67	-0.15	0.28	-0.69 to 0.40	0.60
	mid-Co–B	-0.18	0.53	-1.21 to 0.86	0.74	-0.22	1.0	-2.30 to 1.86	0.83	-0.51	0.52	-1.53 to 0.51	0.33	-0.33	0.35	-1.01 to 0.35	0.34
	mid-Co–Gn	-0.52	0.57	-1.64 to 0.59	0.35	-0.52	1.2	-2.89 to 1.85	0.66	-0.57	0.56	-1.68 to 0.53	0.31	-0.55	0.38	-1.29 to 0.19	0.15
	mid-Go–Gn	-0.40	0.41	-1.21 to 0.41	0.33	-0.41	1.0	-2.41 to 1.59	0.68	-0.36	0.47	-1.29 to 0.58	0.45	-0.38	0.30	-0.96 to 0.20	0.20
	mid-Co–mid-Go	0.13	0.44	-0.73 to 0.99	0.77	-0.31	0.95	-2.19 to 1.58	0.75	0.081	0.46	-0.83 to 1.00	0.86	0.065	0.30	-0.52 to 0.65	0.83
ratio variables	ramus length/SN length	-0.0021	0.0075	-0.017 to 0.013	0.78	0.0019	0.014	-0.026 to 0.030	0.89	0.0083	0.0073	-0.006 to 0.023	0.26	0.0031	0.0049	-0.007 to 0.013	0.53
	mandibular body length/SN length	-0.0062	0.0075	-0.021 to 0.009	0.41	-0.0073	0.015	-0.036 to 0.022	0.62	0.0018	0.0077	-0.013 to 0.017	0.82	-0.0029	0.0050	-0.013 to 0.007	0.56

Category	Measurements	Japan (n=199)				Korea (n=79)				Egypt (n=170)				Fixed-effect meta-analysis			
		B	SE	95% CI	*p*-value	B	SE	95% CI	*p*-value	B	SE	95% CI	*p*-value	B	SE	95% CI	*p*-value
	ramus length/mandibular body length	0.0017	0.0048	-0.008 to 0.011	0.72	0.0049	0.0075	-0.010 to 0.020	0.52	0.0051	0.0057	-0.006 to 0.016	0.37	0.0034	0.0033	-0.003 to 0.010	0.30
	ramus length/ANS-PNS	-0.0085	0.011	-0.029 to 0.012	0.42	-0.0079	0.023	-0.054 to 0.039	0.74	0.018	0.012	-0.006 to 0.041	0.14	0.0019	0.0075	-0.013 to 0.017	0.80
	mid-Co–Gn/ANS–PNS	-0.025	0.018	-0.061 to 0.011	0.18	-0.026	0.039	-0.105 to 0.052	0.51	0.027	0.020	-0.013 to 0.067	0.18	-0.0040	0.013	-0.029 to 0.021	0.76
	mandibular body length/ANS–PNS	-0.016	0.012	-0.040 to 0.007	0.17	-0.027	0.027	-0.082 to 0.028	0.33	0.013	0.014	-0.015 to 0.040	0.35	-0.0062	0.0086	-0.023 to 0.011	0.47
	SN length/Co–Co	0.0023	0.0039	-0.005 to 0.010	0.56	-0.0050	0.0070	-0.019 to 0.009	0.48	-0.0052	0.0052	-0.015 to 0.005	0.32	-0.0011	0.0028	-0.007 to 0.004	0.69
	ramus length/Co–Co	0.0011	0.0045	-0.008 to 0.010	0.81	-0.0023	0.0093	-0.021 to 0.016	0.81	0.0011	0.0053	-0.009 to 0.012	0.84	0.00067	0.0032	-0.006 to 0.007	0.84
	mandibular body length/Co–Co	-0.00069	0.0047	-0.010 to 0.009	0.88	-0.010	0.010	-0.030 to 0.010	0.33	-0.0058	0.0061	-0.018 to 0.006	0.34	-0.0035	0.0035	-0.010 to 0.003	0.32
	ANS–PNS/Co–Co	0.0035	0.0037	-0.004 to 0.011	0.34	0.0010	0.0061	-0.011 to 0.013	0.88	-0.0069	0.0049	-0.017 to 0.003	0.16	-0.000037	0.0027	-0.005 to 0.005	0.99
	Go–Go/Co–Co	-0.0014	0.0053	-0.012 to 0.009	0.80	0.0066	0.011	-0.015 to 0.028	0.55	-0.017	0.0069	-0.030 to -0.003	0.016*	-0.0054	0.0039	-0.013 to 0.002	0.17
	mid-Co–mid-Go/Co–Co	0.0029	0.0049	-0.007 to 0.013	0.55	-0.0057	0.011	-0.027 to 0.015	0.59	0.0018	0.0054	-0.009 to 0.012	0.74	0.0016	0.0034	-0.005 to 0.008	0.65
	mid-Go–Gn/Co–Co	-0.0014	0.0049	-0.011 to 0.008	0.77	-0.0074	0.011	-0.029 to 0.014	0.49	-0.0026	0.0063	-0.015 to 0.010	0.68	-0.0025	0.0036	-0.010 to 0.005	0.49
	mid-Co–B/Co–Co	0.0014	0.0070	-0.012 to 0.015	0.84	-0.0066	0.014	-0.035 to 0.021	0.64	-0.0040	0.0080	-0.020 to 0.012	0.62	-0.0016	0.0049	-0.011 to 0.008	0.74
	mid-Go–B/Co–Co	0.0018	0.0046	-0.007 to 0.011	0.70	-0.0070	0.010	-0.027 to 0.013	0.48	-0.00084	0.0063	-0.013 to 0.012	0.89	-0.00013	0.0035	-0.007 to 0.007	0.97
	mid-Co–Gn/Co–Co	-0.0016	0.0074	-0.016 to 0.013	0.83	-0.010	0.015	-0.041 to 0.021	0.51	-0.0044	0.0085	-0.021 to 0.012	0.61	-0.0037	0.0053	-0.014 to 0.007	0.48
angular measurements	SNA	-0.11	0.35	-0.81 to 0.58	0.75	0.39	0.52	-0.65 to 1.43	0.46	0.78	0.44	-0.09 to 1.66	0.078	0.27	0.24	-0.21 to 0.75	0.27
	SNB	-0.63	0.44	-1.50 to 0.23	0.15	-0.29	0.96	-2.20 to 1.62	0.76	0.50	0.43	-0.35 to 1.35	0.25	-0.080	0.29	-0.65 to 0.49	0.78
	ANB	0.52	0.33	-0.12 to 1.17	0.11	0.69	0.79	-0.89 to 2.26	0.39	0.28	0.29	-0.29 to 0.86	0.33	0.41	0.21	0.00 to 0.82	0.051
	FMA	-0.17	0.61	-1.38 to 1.04	0.78	-0.10	1.1	-2.31 to 2.11	0.93	-0.49	0.59	-1.65 to 0.66	0.40	-0.31	0.40	-1.08 to 0.47	0.44
	S–N–Pog angle	-0.62	0.45	-1.50 to 0.26	0.17	-0.98	1.0	-3.06 to 1.10	0.35	0.37	0.44	-0.50 to 1.24	0.41	-0.19	0.30	-0.78 to 0.40	0.53
	S–N–Pog–SNB difference	0.013	0.22	-0.41 to 0.44	0.95	-0.69	0.36	-1.41 to 0.03	0.060	-0.13	0.16	-0.46 to 0.19	0.42	-0.15	0.12	-0.39 to 0.09	0.22
	Gonial angle (mid-Co–mid-Go–Gn)	-0.11	0.50	-1.11 to 0.88	0.82	0.26	0.95	-1.64 to 2.15	0.79	0.18	0.49	-0.80 to 1.16	0.72	0.063	0.33	-0.59 to 0.71	0.85
	mid-Go–Gn–B	0.71	0.49	-0.26 to 1.68	0.15	-0.15	0.85	-1.84 to 1.54	0.86	0.24	0.54	-0.82 to 1.31	0.65	0.40	0.33	-0.25 to 1.05	0.23
	mid-Go–Pog–B	0.57	0.51	-0.43 to 1.57	0.26	-0.23	0.98	-2.18 to 1.71	0.81	0.26	0.59	-0.91 to 1.43	0.66	0.35	0.36	-0.35 to 1.05	0.33
	mid-Go–Pog–Gn	-0.70	0.73	-2.13 to 0.73	0.33	0.63	1.4	-2.06 to 3.32	0.64	-0.017	0.76	-1.52 to 1.48	0.98	-0.24	0.49	-1.20 to 0.71	0.62
	GoR–Gn–GoL	0.0084	0.40	-0.79 to 0.80	0.98	1.0	0.88	-0.75 to 2.75	0.26	-0.73	0.49	-1.69 to 0.23	0.14	-0.15	0.29	-0.72 to 0.43	0.61

Values are presented as regression coefficients (B), standard errors (SE), unadjusted 95% confidence intervals (CI), and raw *p* values. B represents the estimated change in each outcome per additional derived A allele under an additive genetic model. Cohort-specific models were adjusted for sex and age and, in the Japan-recruited cohort, additionally for imaging modality. Fixed-effect meta-analysis estimates were obtained by inverse-variance weighting of the cohort-specific estimates. An asterisk indicates *nominal raw $P < 0.05$; all P values in this table are

unadjusted, and no association remained statistically significant after Bonferroni or Benjamini–Hochberg false-discovery-rate correction across the complete family of 408 secondary tests. Volume is expressed in mm^3, linear measurements in mm, ratio variables are dimensionless, and angular measurements are expressed in degrees. In the Korea-recruited cohort, the two SNPs had identical derived-allele dosages and consequently yielded identical estimates; these results do not represent independent association signals. PTH1R, parathyroid hormone 1 receptor; SNP, single-nucleotide polymorphism.

Table S3. Genotype distributions according to imaging modality in the Japan-recruited cohort.

SNP	Genotype	CBCT (N=158), n (%)	MDCT (N=41), n (%)	Fisher's exact P
rs724448	C/C	50 (31.6)	14 (34.1)	
	A/C	73 (46.2)	18 (43.9)	0.97
	A/A	35 (22.2)	9 (22.0)	
rs2242116	G/G	54 (34.2)	14 (34.1)	
	A/G	70 (44.3)	18 (43.9)	1.0
	A/A	34 (21.5)	9 (22.0)	

Values are presented as n (%) within each imaging-modality group. The Japan-recruited cohort included 158 participants examined using CBCT and 41 examined using MDCT. Genotype distributions between imaging modalities were compared separately for each SNP using a two-sided Fisher's exact test. The same P value is repeated across the genotype rows because one overall test was performed for each SNP. No statistically significant difference in genotype distribution was detected between CBCT and MDCT for either variant. CBCT, cone-beam computed tomography; MDCT, multidetector computed tomography; SNP, single-nucleotide polymorphism.

Table S4. Japan imaging-modality sensitivity analyses of associations between *PTH1R* variants and mandibular morphology.

Japan-recruited cohort: imaging-modality sensitivity analysis							
SNP	Outcome	Modality-adjusted analysis (N=199)		CBCT-only sensitivity analysis (N=158)			
		B (95% CI)	*p*-value	B (95% CI)	*p*-value	CBCT Bonferroni P	CBCT BH-FDR P
rs724448	PC1	-0.0020 (-0.0065, 0.0025)	0.380	0.0010 (-0.0038, 0.0058)	0.678	1.000	0.868
	PC2	0.0018 (-0.0024, 0.0059)	0.400	0.0021 (-0.0026, 0.0067)	0.377	1.000	0.814
	PC3	0.0003 (-0.0031, 0.0037)	0.858	0.0000 (-0.0038, 0.0039)	0.988	1.000	0.988
	PC4	0.0002 (-0.0025, 0.0028)	0.899	0.0009 (-0.0021, 0.0039)	0.567	1.000	0.854
	PC5	-0.0023 (-0.0046, 0.0000)	0.053	-0.0028 (-0.0054, -0.0002)	0.036*	1.000	0.804
	PC6	-0.0003 (-0.0026, 0.0021)	0.826	0.0000 (-0.0025, 0.0025)	0.980	1.000	0.988
	PC7	0.0017 (-0.0004, 0.0038)	0.104	0.0029 (0.0009, 0.0050)	0.006*	0.576	0.487
	PC8	-0.0002 (-0.0019, 0.0014)	0.792	0.0004 (-0.0015, 0.0023)	0.698	1.000	0.868
	PC9	-0.0004 (-0.0023, 0.0014)	0.631	-0.0006 (-0.0027, 0.0014)	0.548	1.000	0.854
	PC10	0.0007 (-0.0009, 0.0022)	0.405	0.0012 (-0.0006, 0.0029)	0.181	1.000	0.804

SNP	Outcome	Modality-adjusted analysis (N=199)		CBCT-only sensitivity analysis (N=158)			
		B (95% CI)	*p*-value	B (95% CI)	*p*-value	CBCT Bonferroni P	CBCT BH-FDR P
	PC11	0.0004 (-0.0011, 0.0020)	0.564	0.0004 (-0.0013, 0.0022)	0.629	1.000	0.854
	ln (centroid size)	-0.0038 (-0.0104, 0.0027)	0.247	-0.0036 (-0.0107, 0.0035)	0.319	1.000	0.814
	Volume	-107.4815 (-1279.7209, 1064.7578)	0.857	-185.0722 (-1460.2785, 1090.1341)	0.775	1.000	0.930
	SN length	0.1235 (-0.4444, 0.6914)	0.668	0.1411 (-0.4976, 0.7798)	0.663	1.000	0.867
	ramus length	-0.1504 (-0.9645, 0.6637)	0.716	0.2299 (-0.6031, 1.0630)	0.586	1.000	0.854
	mandibular body length	-0.2560 (-1.0186, 0.5066)	0.509	-0.2177 (-1.0434, 0.6079)	0.603	1.000	0.854
	ANS-PNS	0.1707 (-0.4084, 0.7498)	0.562	0.1895 (-0.4641, 0.8431)	0.568	1.000	0.854
	intergonial width (Go-Go)	-0.2261 (-1.3333, 0.8810)	0.688	-0.4807 (-1.7271, 0.7657)	0.447	1.000	0.814
	intercondylar width (Co-Co)	-0.2235 (-1.2305, 0.7836)	0.662	-0.8637 (-1.9919, 0.2645)	0.133	1.000	0.804
	mid-Go-B	-0.1194 (-0.8765, 0.6376)	0.756	0.1008 (-0.7211, 0.9227)	0.809	1.000	0.942
	mid-Co–B	-0.2184 (-1.2633, 0.8266)	0.681	0.3170 (-0.7357, 1.3698)	0.553	1.000	0.854
	mid-Co–Gn	-0.4723 (-1.5989, 0.6543)	0.409	0.0688 (-1.0629, 1.2004)	0.905	1.000	0.942
	mid-Go–Gn	-0.3518 (-1.1723, 0.4688)	0.399	-0.1537 (-1.0166, 0.7091)	0.725	1.000	0.891
	mid-Co–mid-Go	0.0539 (-0.8121, 0.9199)	0.902	0.4091 (-0.4618, 1.2799)	0.355	1.000	0.814
	ramus length/SN length	-0.0042 (-0.0192, 0.0108)	0.584	0.0013 (-0.0147, 0.0173)	0.874	1.000	0.942
	mandibular body length/SN length	-0.0062 (-0.0210, 0.0087)	0.415	-0.0063 (-0.0231, 0.0106)	0.463	1.000	0.814
	ramus length/mandibular body length	0.0001 (-0.0094, 0.0095)	0.991	0.0041 (-0.0060, 0.0141)	0.425	1.000	0.814
	ramus length/ANS-PNS	-0.0085 (-0.0295, 0.0126)	0.429	-0.0022 (-0.0255, 0.0211)	0.851	1.000	0.942
	mid-Co–Gn/ANS–PNS	-0.0204 (-0.0568, 0.0161)	0.272	-0.0152 (-0.0551, 0.0246)	0.451	1.000	0.814
	mandibular body length/ANS–PNS	-0.0122 (-0.0359, 0.0116)	0.313	-0.0128 (-0.0387, 0.0131)	0.331	1.000	0.814
	SN length/Co–Co	0.0024 (-0.0053, 0.0101)	0.540	0.0064 (-0.0021, 0.0149)	0.140	1.000	0.804
	ramus length/Co–Co	-0.0001 (-0.0092, 0.0089)	0.976	0.0069 (-0.0027, 0.0165)	0.157	1.000	0.804
	mandibular body length/Co–Co	-0.0006 (-0.0099, 0.0087)	0.907	0.0049 (-0.0053, 0.0151)	0.340	1.000	0.814
	ANS–PNS/Co–Co	0.0026 (-0.0049, 0.0100)	0.494	0.0056 (-0.0027, 0.0139)	0.182	1.000	0.804
	Go–Go/Co–Co	-0.0001 (-0.0107, 0.0105)	0.987	0.0032 (-0.0087, 0.0150)	0.598	1.000	0.854
	mid-Co–mid-Go/Co–Co	0.0017 (-0.0080, 0.0114)	0.735	0.0085 (-0.0018, 0.0187)	0.104	1.000	0.804
	mid-Go–Gn/Co–Co	-0.0017 (-0.0114, 0.0080)	0.734	0.0046 (-0.0058, 0.0150)	0.379	1.000	0.814

Japan-recruited cohort: imaging-modality sensitivity analysis							
SNP	Outcome	Modality-adjusted analysis (N=199)		CBCT-only sensitivity analysis (N=158)			
		B (95% CI)	*p*-value	B (95% CI)	*p*-value	CBCT Bonferroni P	CBCT BH-FDR P
	mid-Co–B/Co–Co	0.0001 (-0.0139, 0.0141)	0.989	0.0115 (-0.0036, 0.0265)	0.134	1.000	0.804
	mid-Go–B/Co–Co	0.0006 (-0.0087, 0.0098)	0.901	0.0068 (-0.0033, 0.0170)	0.187	1.000	0.804
	mid-Co–Gn/Co–Co	-0.0022 (-0.0170, 0.0127)	0.773	0.0100 (-0.0058, 0.0258)	0.215	1.000	0.804
	SNA	-0.1789 (-0.8806, 0.5227)	0.616	-0.0286 (-0.7817, 0.7245)	0.940	1.000	0.969
	SNB	-0.6197 (-1.4915, 0.2521)	0.163	-0.3408 (-1.2400, 0.5584)	0.455	1.000	0.814
	ANB	0.4408 (-0.2113, 1.0928)	0.184	0.3122 (-0.3834, 1.0079)	0.377	1.000	0.814
	FMA	0.0254 (-1.1952, 1.2459)	0.967	-0.0849 (-1.4261, 1.2563)	0.901	1.000	0.942
	S–N–Pog angle	-0.5869 (-1.4785, 0.3047)	0.196	-0.3767 (-1.3240, 0.5706)	0.433	1.000	0.814
	S–N–Pog–SNB difference	0.0328 (-0.3983, 0.4639)	0.881	-0.0359 (-0.5428, 0.4710)	0.889	1.000	0.942
	Gonial angle (mid-Co–mid-Go–Gn)	-0.0478 (-1.0518, 0.9562)	0.925	-0.2605 (-1.3456, 0.8246)	0.636	1.000	0.854
	mid-Go–Gn–B	0.4875 (-0.4921, 1.4671)	0.328	0.5321 (-0.5483, 1.6125)	0.332	1.000	0.814
	mid-Go–Pog–B	0.4037 (-0.6061, 1.4135)	0.431	0.3887 (-0.6956, 1.4729)	0.480	1.000	0.830
	mid-Go–Pog–Gn	-0.4267 (-1.8729, 1.0195)	0.561	-0.7012 (-2.4076, 1.0052)	0.418	1.000	0.814
	GoR–Gn–GoL	0.0971 (-0.7057, 0.8999)	0.812	-0.1712 (-1.0263, 0.6838)	0.693	1.000	0.868
rs2242116	PC1	-0.0017 (-0.0061, 0.0028)	0.468	0.0014 (-0.0033, 0.0061)	0.562	1.000	0.854
	PC2	0.0028 (-0.0013, 0.0069)	0.176	0.0034 (-0.0012, 0.0080)	0.144	1.000	0.804
	PC3	0.0001 (-0.0033, 0.0035)	0.971	-0.0003 (-0.0041, 0.0035)	0.892	1.000	0.942
	PC4	0.0007 (-0.0020, 0.0033)	0.613	0.0015 (-0.0015, 0.0045)	0.318	1.000	0.814
	PC5	-0.0025 (-0.0048, -0.0002)	0.031*	-0.0030 (-0.0056, -0.0005)	0.020*	1.000	0.686
	PC6	-0.0004 (-0.0027, 0.0019)	0.721	-0.0002 (-0.0027, 0.0023)	0.893	1.000	0.942
	PC7	0.0016 (-0.0005, 0.0037)	0.132	0.0027 (0.0007, 0.0048)	0.010*	0.974	0.487
	PC8	-0.0002 (-0.0018, 0.0014)	0.840	0.0004 (-0.0015, 0.0023)	0.659	1.000	0.867
	PC9	-0.0006 (-0.0025, 0.0012)	0.479	-0.0009 (-0.0029, 0.0011)	0.392	1.000	0.814
	PC10	0.0005 (-0.0011, 0.0020)	0.554	0.0009 (-0.0008, 0.0026)	0.288	1.000	0.814
	PC11	0.0005 (-0.0010, 0.0020)	0.528	0.0005 (-0.0013, 0.0022)	0.592	1.000	0.854
	ln (centroid size)	-0.0048 (-0.0113, 0.0017)	0.145	-0.0048 (-0.0118, 0.0022)	0.180	1.000	0.804
	Volume	-128.9935 (-1290.5421, 1032.5550)	0.827	-208.9573 (-1470.3974, 1052.4828)	0.744	1.000	0.903

Japan-recruited cohort: imaging-modality sensitivity analysis							
SNP	Outcome	Modality-adjusted analysis (N=199)		CBCT-only sensitivity analysis (N=158)			
		B (95% CI)	*p*-value	B (95% CI)	*p*-value	CBCT Bonferroni P	CBCT BH-FDR P
	SN length	0.0533 (-0.5096, 0.6163)	0.852	0.0488 (-0.5834, 0.6810)	0.879	1.000	0.942
	ramus length	-0.0790 (-0.8859, 0.7279)	0.847	0.3139 (-0.5095, 1.1373)	0.453	1.000	0.814
	mandibular body length	-0.3560 (-1.1109, 0.3988)	0.353	-0.3446 (-1.1602, 0.4711)	0.405	1.000	0.814
	ANS-PNS	0.2297 (-0.3437, 0.8032)	0.430	0.2634 (-0.3825, 0.9094)	0.422	1.000	0.814
	intergonial width (Go-Go)	-0.4508 (-1.5465, 0.6449)	0.418	-0.7592 (-1.9886, 0.4702)	0.224	1.000	0.804
	intercondylar width (Co-Co)	-0.3186 (-1.3160, 0.6788)	0.529	-0.9705 (-2.0842, 0.1432)	0.087	1.000	0.804
	mid-Go-B	-0.0633 (-0.8136, 0.6871)	0.868	0.1677 (-0.6451, 0.9805)	0.684	1.000	0.868
	mid-Co–B	-0.1764 (-1.2120, 0.8593)	0.737	0.3606 (-0.6805, 1.4017)	0.495	1.000	0.841
	mid-Co–Gn	-0.5250 (-1.6409, 0.5909)	0.355	-0.0113 (-1.1309, 1.1083)	0.984	1.000	0.988
	mid-Go–Gn	-0.3992 (-1.2119, 0.4134)	0.334	-0.2193 (-1.0725, 0.6340)	0.612	1.000	0.854
	mid-Co–mid-Go	0.1291 (-0.7289, 0.9871)	0.767	0.4985 (-0.3618, 1.3587)	0.254	1.000	0.814
	ramus length/SN length	-0.0021 (-0.0170, 0.0128)	0.779	0.0038 (-0.0120, 0.0197)	0.631	1.000	0.854
	mandibular body length/SN length	-0.0062 (-0.0209, 0.0085)	0.409	-0.0062 (-0.0229, 0.0104)	0.461	1.000	0.814
	ramus length/mandibular body length	0.0017 (-0.0077, 0.0111)	0.724	0.0060 (-0.0038, 0.0159)	0.229	1.000	0.804
	ramus length/ANS-PNS	-0.0085 (-0.0294, 0.0124)	0.422	-0.0024 (-0.0254, 0.0207)	0.839	1.000	0.942
	mid-Co–Gn/ANS–PNS	-0.0247 (-0.0607, 0.0114)	0.178	-0.0208 (-0.0602, 0.0186)	0.298	1.000	0.814
	mandibular body length/ANS–PNS	-0.0163 (-0.0397, 0.0072)	0.173	-0.0179 (-0.0435, 0.0076)	0.168	1.000	0.804
	SN length/Co–Co	0.0023 (-0.0053, 0.0099)	0.557	0.0061 (-0.0023, 0.0146)	0.153	1.000	0.804
	ramus length/Co–Co	0.0011 (-0.0078, 0.0100)	0.809	0.0084 (-0.0011, 0.0178)	0.083	1.000	0.804
	mandibular body length/Co–Co	-0.0007 (-0.0099, 0.0085)	0.883	0.0047 (-0.0054, 0.0148)	0.363	1.000	0.814
	ANS–PNS/Co–Co	0.0035 (-0.0038, 0.0109)	0.344	0.0068 (-0.0014, 0.0149)	0.104	1.000	0.804
	Go–Go/Co–Co	-0.0014 (-0.0119, 0.0092)	0.801	0.0015 (-0.0102, 0.0132)	0.800	1.000	0.942
	mid-Co–mid-Go/Co–Co	0.0029 (-0.0067, 0.0125)	0.551	0.0099 (-0.0002, 0.0200)	0.054	1.000	0.804
	mid-Go–Gn/Co–Co	-0.0014 (-0.0111, 0.0082)	0.770	0.0048 (-0.0055, 0.0151)	0.356	1.000	0.814
	mid-Co–B/Co–Co	0.0014 (-0.0124, 0.0152)	0.841	0.0129 (-0.0019, 0.0277)	0.087	1.000	0.804
	mid-Go–B/Co–Co	0.0018 (-0.0074, 0.0109)	0.705	0.0082 (-0.0018, 0.0182)	0.109	1.000	0.804
	mid-Co–Gn/Co–Co	-0.0016 (-0.0163, 0.0131)	0.827	0.0104 (-0.0052, 0.0260)	0.191	1.000	0.804

Japan-recruited cohort: imaging-modality sensitivity analysis							
SNP	Outcome	Modality-adjusted analysis (N=199)		CBCT-only sensitivity analysis (N=158)			
		B (95% CI)	*p*-value	B (95% CI)	*p*-value	CBCT Bonferroni P	CBCT BH-FDR P
	SNA	-0.1110 (-0.8066, 0.5846)	0.753	0.0556 (-0.6894, 0.8006)	0.883	1.000	0.942
	SNB	-0.6317 (-1.4954, 0.2319)	0.151	-0.3654 (-1.2546, 0.5239)	0.418	1.000	0.814
	ANB	0.5207 (-0.1242, 1.1656)	0.113	0.4210 (-0.2657, 1.1076)	0.228	1.000	0.804
	FMA	-0.1696 (-1.3788, 1.0396)	0.782	-0.3255 (-1.6514, 1.0004)	0.628	1.000	0.854
	S–N–Pog angle	-0.6190 (-1.5020, 0.2640)	0.168	-0.4242 (-1.3608, 0.5124)	0.372	1.000	0.814
	S–N–Pog–SNB difference	0.0127 (-0.4145, 0.4399)	0.953	-0.0589 (-0.5603, 0.4426)	0.817	1.000	0.942
	Gonial angle (mid-Co–mid-Go–Gn)	-0.1119 (-1.1067, 0.8829)	0.825	-0.3428 (-1.4157, 0.7301)	0.529	1.000	0.854
	mid-Go–Gn–B	0.7114 (-0.2565, 1.6793)	0.149	0.8162 (-0.2480, 1.8804)	0.132	1.000	0.804
	mid-Go–Pog–B	0.5701 (-0.4289, 1.5691)	0.262	0.5992 (-0.4709, 1.6693)	0.270	1.000	0.814
	mid-Go–Pog–Gn	-0.7041 (-2.1350, 0.7267)	0.333	-1.0494 (-2.7328, 0.6341)	0.220	1.000	0.804
	GoR–Gn–GoL	0.0084 (-0.7872, 0.8041)	0.983	-0.2763 (-1.1215, 0.5688)	0.519	1.000	0.854

Values are presented as regression coefficients (B), unadjusted 95% confidence intervals (CIs), and raw P values unless otherwise indicated. B represents the estimated change in each outcome per additional derived A allele. The modality-adjusted analysis included all 199 participants in the Japan-recruited cohort and was adjusted for sex, age, and imaging modality (CBCT = 0; MDCT = 1). The CBCT-only sensitivity analysis included 158 participants and was adjusted for sex and age. Full-Japan results were included in the complete 408-test secondary family; their Bonferroni- and BH-FDR-adjusted *p* values are reported in Table S7. For the CBCT-only sensitivity analysis, Bonferroni and BH-FDR adjustments were calculated across a separate family of 102 tests (51 outcomes x 2 SNPs). An asterisk indicates nominal raw $p < 0.05$. No CBCT-only association remained statistically significant after either correction. ln(centroid size) was analyzed on the natural-log scale. PTH1R, parathyroid hormone 1 receptor; SNP, single-nucleotide polymorphism; CBCT, cone-beam computed tomography; MDCT, multidetector computed tomography; BH-FDR, Benjamini-Hochberg false-discovery rate; PC, principal component.

Table S5. Between-cohort heterogeneity and random-effects sensitivity analyses for associations of PTH1R variants with mandibular morphological outcomes.

SNP	Outcome group	Outcome	Cochran's Q	Phet	I^2 (%)	Bonferroni Phet	BH-FDR Phet	Random effects B	Random effects SE	Random effects 95% CI
rs724448	Volume	Volume	0.13	0.94	0.0	1.0	0.98	-210	411	-1014 to 595
	Linear measurement	SN Length	4.2	0.12	53	1.0	0.78	-0.28	0.31	-0.89 to 0.33
		ramus length	0.14	0.93	0.0	1.0	0.98	-0.17	0.29	-0.74 to 0.40
		mandibular body length	1.9	0.39	0.0	1.0	0.98	-0.61	0.27	-1.15 to -0.08
		ANS-PNS	7.1	0.029	72	1.0	0.43	-0.23	0.46	-1.14 to 0.68
		intergonial width (Go-Go)	7.1	0.029	72	1.0	0.43	-0.32	0.75	-1.80 to 1.15

SNP	Outcome group	Outcome	Cochran's Q	Phet	I² (%)	Bonferroni Phet	BH-FDR Phet	Random effects B	Random effects SE	Random effects 95% CI
		intercondylar width (Co-Co)	0.76	0.68	0.0	1.0	0.98	0.041	0.36	-0.67 to 0.75
		mid-Go-B	1.1	0.57	0.0	1.0	0.98	-0.38	0.28	-0.93 to 0.17
		mid-Co–B	2.3	0.31	14	1.0	0.91	-0.69	0.39	-1.45 to 0.07
		mid-Co–Gn	1.1	0.58	0.0	1.0	0.98	-0.84	0.38	-1.59 to -0.10
		mid-Go–Gn	0.71	0.70	0.0	1.0	0.98	-0.56	0.30	-1.15 to 0.03
		mid-Co–mid-Go	0.15	0.93	0.0	1.0	0.98	-0.054	0.30	-0.65 to 0.54
	Ratio measurement	ramus length/SN length	0.97	0.62	0.0	1.0	0.98	0.0013	0.0050	-0.008 to 0.011
		mandibular body length/SN length	0.32	0.85	0.0	1.0	0.98	-0.0040	0.0051	-0.014 to 0.006
		ramus length/mandibular body length	0.51	0.77	0.0	1.0	0.98	0.0025	0.0033	-0.004 to 0.009
		ramus length/ANS-PNS	3.3	0.19	39	1.0	0.82	0.0027	0.010	-0.018 to 0.023
		mid-Co–Gn/ANS–PNS	4.0	0.13	50	1.0	0.78	-0.0011	0.020	-0.040 to 0.038
		mandibular body length/ANS–PNS	3.3	0.19	39	1.0	0.82	-0.0032	0.012	-0.027 to 0.020
		SN length/Co–Co	3.2	0.20	38	1.0	0.82	-0.0029	0.0038	-0.010 to 0.005
		ramus length/Co–Co	0.25	0.88	0.0	1.0	0.98	-0.0017	0.0033	-0.008 to 0.005
		mandibular body length/Co–Co	2.5	0.29	18	1.0	0.91	-0.0059	0.0041	-0.014 to 0.002
		ANS–PNS/Co–Co	5.3	0.069	63	1.0	0.70	-0.0025	0.0046	-0.012 to 0.007
		Go–Go/Co–Co	5.6	0.062	64	1.0	0.70	-0.0050	0.0072	-0.019 to 0.009
		mid-Co–mid-Go/Co–Co	0.51	0.78	0.0	1.0	0.98	-0.00058	0.0035	-0.007 to 0.006
		mid-Go–Gn/Co–Co	1.2	0.56	0.0	1.0	0.98	-0.0051	0.0037	-0.012 to 0.002
		mid-Co–B/Co–Co	1.9	0.38	0.0	1.0	0.98	-0.0064	0.0050	-0.016 to 0.003
		mid-Go–B/Co–Co	1.6	0.46	0.0	1.0	0.98	-0.0032	0.0035	-0.010 to 0.004
		mid-Co–Gn/Co–Co	1.3	0.53	0.0	1.0	0.98	-0.0079	0.0053	-0.018 to 0.002
	Angular measurement	SNA	0.97	0.62	0.0	1.0	0.98	0.064	0.25	-0.42 to 0.55
		SNB	0.78	0.68	0.0	1.0	0.98	-0.34	0.30	-0.92 to 0.24
		ANB	0.29	0.86	0.0	1.0	0.98	0.38	0.21	-0.04 to 0.79
		FMA	0.40	0.82	0.0	1.0	0.98	-0.23	0.40	-1.02 to 0.56
		S–N–Pog angle	0.70	0.70	0.0	1.0	0.98	-0.44	0.31	-1.04 to 0.16
		S–N–Pog–SNB difference	3.0	0.23	32	1.0	0.82	-0.16	0.16	-0.47 to 0.16
		Gonial angle (mid-Co–mid-Go–Gn)	0.080	0.96	0.0	1.0	0.99	0.014	0.34	-0.64 to 0.67
		mid-Go–Gn–B	0.45	0.80	0.0	1.0	0.98	0.28	0.34	-0.39 to 0.94

SNP	Outcome group	Outcome	Cochran's Q	Phet	I² (%)	Bonferroni Phet	BH-FDR Phet	Random effects B	Random effects SE	Random effects 95% CI
		mid-Go–Pog–B	0.34	0.85	0.0	1.0	0.98	0.26	0.36	-0.45 to 0.97
		mid-Go–Pog–Gn	0.48	0.79	0.0	1.0	0.98	-0.22	0.50	-1.19 to 0.75
		GoR–Gn–GoL	1.8	0.41	0.0	1.0	0.98	0.048	0.30	-0.53 to 0.63
	GPA/PCA	PC1	0.13	0.93	0.0	1.0	0.98	-0.0022	0.0016	-0.0054 to 0.0010
		PC2	0.18	0.91	0.0	1.0	0.98	0.0018	0.0014	-0.0010 to 0.0046
		PC3	11	0.0044	82	0.4	0.43	-0.0011	0.0027	-0.0065 to 0.0042
		PC4	0.11	0.94	0.0	1.0	0.98	0.00036	0.0010	-0.0015 to 0.0022
		PC5	4.5	0.10	56	1.0	0.78	-0.00036	0.0014	-0.0031 to 0.0023
		PC6	0.53	0.77	0.0	1.0	0.98	0.00023	0.00081	-0.0014 to 0.0018
		PC7	4.1	0.13	51	1.0	0.78	0.00066	0.0011	-0.0016 to 0.0029
		PC8	5.1	0.078	61	1.0	0.72	0.00082	0.0011	-0.0013 to 0.0030
		PC9	8.1	0.017	75	1.0	0.43	0.00080	0.0014	-0.0019 to 0.0035
		PC10	0.14	0.93	0.0	1.0	0.98	0.00077	0.00059	-0.0004 to 0.0019
		PC11	0.47	0.79	0.0	1.0	0.98	0.00068	0.00054	-0.0004 to 0.0017
		ln (centroid size)	1.1	0.58	0.0	1.0	0.98	-0.0053	0.0023	-0.0099 to -0.0007
rs2242116	Volume	Volume	0.30	0.86	0.0	1.0	0.98	67	405	-727 to 861
	Linear measurement	SN Length	2.34	0.31	14	1.0	0.91	-0.23	0.22	-0.67 to 0.20
		ramus length	0.031	0.98	0.0	1.0	1.0	-0.031	0.29	-0.59 to 0.53
		mandibular body length	0.36	0.84	0.0	1.0	0.98	-0.52	0.27	-1.05 to 0.01
		ANS-PNS	4.7	0.10	57	1.0	0.78	-0.10	0.37	-0.83 to 0.63
		intergonial width (Go-Go)	7.5	0.023	73	1.0	0.43	-0.44	0.76	-1.94 to 1.06
		intercondylar width (Co-Co)	0.85	0.65	0.0	1.0	0.98	-0.12	0.36	-0.82 to 0.58
		mid-Go-B	0.15	0.93	0.0	1.0	0.98	-0.15	0.28	-0.69 to 0.40
		mid-Co–B	0.22	0.90	0.0	1.0	0.98	-0.33	0.35	-1.01 to 0.35
		mid-Co–Gn	0.0043	1.0	0.0	1.0	1.0	-0.55	0.38	-1.29 to 0.19
		mid-Go–Gn	0.0057	1.0	0.0	1.0	1.0	-0.38	0.30	-0.96 to 0.20
		mid-Co–mid-Go	0.18	0.92	0.0	1.0	0.98	0.065	0.30	-0.52 to 0.65
	Ratio measurement	ramus length/SN length	0.99	0.61	0.0	1.0	0.98	0.0031	0.0049	-0.007 to 0.013
		mandibular body length/SN length	0.65	0.72	0.0	1.0	0.98	-0.0029	0.0050	-0.013 to 0.007
		ramus length/mandibular body length	0.26	0.88	0.0	1.0	0.98	0.0034	0.0033	-0.003 to 0.010

SNP	Outcome group	Outcome	Cochran's Q	Phet	I² (%)	Bonferroni Phet	BH-FDR Phet	Random effects B	Random effects SE	Random effects 95% CI
		ramus length/ANS-PNS	2.9	0.23	31	1.0	0.82	0.0020	0.0095	-0.017 to 0.021
		mid-Co–Gn/ANS–PNS	4.0	0.14	50	1.0	0.78	-0.0045	0.019	-0.043 to 0.034
		mandibular body length/ANS–PNS	3.2	0.21	37	1.0	0.82	-0.0064	0.012	-0.029 to 0.016
		SN length/Co–Co	1.7	0.43	0.0	1.0	0.98	-0.0011	0.0028	-0.007 to 0.004
		ramus length/Co–Co	0.11	0.94	0.0	1.0	0.98	0.00067	0.0032	-0.006 to 0.007
		mandibular body length/Co–Co	0.92	0.63	0.0	1.0	0.98	-0.0035	0.0035	-0.010 to 0.003
		ANS–PNS/Co–Co	2.9	0.23	31	1.0	0.82	-0.00038	0.0033	-0.007 to 0.006
		Go–Go/Co–Co	4.5	0.11	55	1.0	0.78	-0.0051	0.0063	-0.018 to 0.007
		mid-Co–mid-Go/Co–Co	0.55	0.76	0.0	1.0	0.98	0.0016	0.0034	-0.005 to 0.008
		mid-Go–Gn/Co–Co	0.26	0.88	0.0	1.0	0.98	-0.0025	0.0036	-0.010 to 0.005
		mid-Co–B/Co–Co	0.40	0.82	0.0	1.0	0.98	-0.0016	0.0049	-0.011 to 0.008
		mid-Go–B/Co–Co	0.66	0.72	0.0	1.0	0.98	-0.00013	0.0035	-0.007 to 0.007
		mid-Co–Gn/Co–Co	0.26	0.88	0.0	1.0	0.98	-0.0037	0.0053	-0.014 to 0.007
	Angular measurement	SNA	2.6	0.28	22	1.0	0.91	0.29	0.28	-0.26 to 0.84
		SNB	3.4	0.18	42	1.0	0.82	-0.10	0.41	-0.91 to 0.71
		ANB	0.42	0.81	0.0	1.0	0.98	0.41	0.21	0.00 to 0.82
		FMA	0.19	0.91	0.0	1.0	0.98	-0.31	0.40	-1.08 to 0.47
		S–N–Pog angle	3.1	0.21	35	1.0	0.82	-0.23	0.40	-1.02 to 0.56
		S–N–Pog–SNB difference	2.8	0.25	29	1.0	0.84	-0.17	0.15	-0.47 to 0.13
		Gonial angle (mid-Co–mid-Go–Gn)	0.22	0.90	0.0	1.0	0.98	0.063	0.33	-0.59 to 0.71
		mid-Go–Gn–B	0.91	0.64	0.0	1.0	0.98	0.40	0.33	-0.25 to 1.05
		mid-Go–Pog–B	0.57	0.75	0.0	1.0	0.98	0.35	0.36	-0.35 to 1.05
		mid-Go–Pog–Gn	0.91	0.64	0.0	1.0	0.98	-0.24	0.49	-1.20 to 0.71
		GoR–Gn–GoL	3.3	0.19	39	1.0	0.82	-0.10	0.40	-0.89 to 0.68
	GPA/PCA	PC1	1.3	0.53	0.0	1.0	0.98	-0.00071	0.0016	-0.0039 to 0.0025
		PC2	0.31	0.86	0.0	1.0	0.98	0.0023	0.0014	-0.0004 to 0.0051
		PC3	9.2	0.010	78	1.0	0.43	-0.0015	0.0025	-0.0064 to 0.0034
		PC4	0.31	0.86	0.0	1.0	0.98	0.00085	0.00094	-0.0010 to 0.0027
		PC5	2.5	0.29	20	1.0	0.91	-0.0012	0.0010	-0.0031 to 0.0008
		PC6	1.5	0.48	0.0	1.0	0.98	0.00042	0.00080	-0.0011 to 0.0020

SNP	Outcome group	Outcome	Cochran's Q	Phet	I² (%)	Bonferroni Phet	BH-FDR Phet	Random effects B	Random effects SE	Random effects 95% CI
		PC7	3.3	0.19	40	1.0	0.82	0.00067	0.0010	-0.0013 to 0.0027
		PC8	5.5	0.063	64	1.0	0.70	0.00089	0.0011	-0.0013 to 0.0031
		PC9	8.1	0.017	75	1.0	0.43	0.00084	0.0014	-0.0019 to 0.0035
		PC10	1.1	0.57	0.0	1.0	0.98	0.00029	0.00058	-0.0009 to 0.0014
		PC11	0.45	0.80	0.0	1.0	0.98	0.00069	0.00054	-0.0004 to 0.0017
		ln (centroid size)	0.55	0.76	0.0	1.0	0.98	-0.0054	0.0023	-0.0100 to -0.0009

Between-cohort heterogeneity was evaluated using Cochran's Q test with 2 degrees of freedom and quantified using I^2. Raw heterogeneity P values, Bonferroni-adjusted heterogeneity *p* values, and BH-FDR-adjusted heterogeneity *p* values are reported. Both multiple-testing procedures were applied across the separate family of 102 SNP–outcome heterogeneity tests; the corresponding Bonferroni threshold was $0.05/102 = 4.90 \times 10^{-4}$. None of the heterogeneity tests remained statistically significant after either correction. Random-effects summary estimates were calculated using the DerSimonian–Laird estimator and are presented as sensitivity analyses to assess dependence of the combined results on the modeling of between-cohort heterogeneity. Random-effects B values represent the estimated change in each outcome per additional derived A allele. With only three cohorts, estimates of between-cohort heterogeneity are imprecise and should be interpreted cautiously. PTH1R, parathyroid hormone 1 receptor; SNP, single-nucleotide polymorphism; B, regression coefficient; SE, standard error; CI, confidence interval; Phet, heterogeneity P value; I^2, percentage of total variation attributed to between-cohort heterogeneity; BH-FDR, Benjamini–Hochberg false-discovery rate; PC, principal component.

Table S6. Intra-observer repeatability of whole mandibular shape, natural-log-transformed centroid size, and principal component scores.

A. Repeatability of centroid size and principal component scores

Outcome	N	ICC(A,1)	95% CI
ln (centroid size)	30	0.99	0.94–0.99
PC1	30	0.96	0.71–0.99
PC2	30	0.91	0.73–0.96
PC3	30	0.73	0.45–0.87
PC4	30	0.35	0.011–0.62
PC5	30	0.56	-0.044–0.82
PC6	30	0.59	0.30–0.78
PC7	30	0.86	0.72–0.93
PC8	30	0.73	0.51–0.86
PC9	30	0.79	0.50–0.90
PC10	30	0.30	-0.073–0.59
PC11	30	0.43	-0.076–0.73

B. Whole-shape repeatability

**Measure**	**Value**

Participants, n	30
Digitizations per participant, n	2
Analytical landmarks and semi-landmarks, n	353
Whole-shape repeatability, R	0.504
95% bootstrap confidence interval	0.390–0.592
Within-participant variation, % of total Procrustes SS	25.42

C. Whole-shape Procrustes ANOVA

**Source**	**df**	**SS**	**MS**	**F**
Between participants	29	0.20	0.0068	3.04
Within participants	30	0.067	0.0022	—
Total	59	0.26	—	—

Thirty participants were randomly selected and their mandibular meshes were redigitized by the same examiner at least two weeks after the initial digitization. All analyses used 353 analytical landmarks and semi-landmarks after exclusion of the Gnathion helper point. A: Outcome-specific repeatability was assessed using ICC(A,1), a two-way random-effects, absolute-agreement, single-measure intraclass correlation coefficient, with 95% CIs. Centroid size was natural-log transformed, and repeated configurations were projected onto the PC axes from the original 448-participant analysis. B–C: Whole-shape repeatability (R) was estimated from a one-way Procrustes ANOVA after joint GPA alignment of all 60 configurations. The 95% percentile bootstrap CI was based on 5,000 paired-participant resamples with the fitted GPA alignment held fixed. Whole-shape R is distinct from the outcome-specific ICC(A,1). Within-participant variation is expressed as a percentage of total Procrustes SS. Degrees of freedom are based on participants and repeated observations; F is descriptive, without a parametric P value. These analyses assess repeat digitization and subsequent shape processing on existing meshes, not independent segmentation or cross-modality reproducibility. CI, confidence interval; df, degrees of freedom; GPA, generalized Procrustes analysis; ICC, intraclass correlation coefficient; MS, mean square; PC, principal component; SS, sum of squares.

Table S7. Raw and multiple-testing-adjusted P values for the 408 secondary univariate association tests of PTH1R variants with mandibular morphological outcomes.

Outcome variable	Japan (n = 199)			Korea (n = 79)			Egypt (n = 170)			Fixed-effect meta-analysis		
	p-value	Bonferroni P	BH-FDR P	*p*-value	Bonferroni P	BH-FDR P	*p*-value	Bonferroni P	BH-FDR P	*p*-value	Bonferroni P	BH-FDR P
rs724448												
Volume	0.86	1.00	0.98	0.97	1.00	0.99	0.55	1.00	0.98	0.61	1.00	0.98
SN length	0.67	1.00	0.98	0.72	1.00	0.98	0.020	1.00	0.54	0.21	1.00	0.98
ramus length	0.72	1.00	0.98	0.94	1.00	0.99	0.56	1.00	0.98	0.56	1.00	0.98
mandibular body length	0.51	1.00	0.98	0.48	1.00	0.98	0.016	1.00	0.51	0.025	1.00	0.54
ANS-PNS	0.56	1.00	0.98	0.59	1.00	0.98	0.0086	1.00	0.49	0.43	1.00	0.98
intergonial width (Go-Go)	0.69	1.00	0.98	0.18	1.00	0.98	0.012	1.00	0.51	0.25	1.00	0.98

Outcome variable	Japan (n = 199)			Korea (n = 79)			Egypt (n = 170)			Fixed-effect meta-analysis		
	p-value	Bonferroni P	BH-FDR P	*p*-value	Bonferroni P	BH-FDR P	*p*-value	Bonferroni P	BH-FDR P	*p*-value	Bonferroni P	BH-FDR P
intercondylar width (Co-Co)	0.66	1.00	0.98	0.48	1.00	0.98	0.79	1.00	0.98	0.91	1.00	0.98
mid-Go-B	0.76	1.00	0.98	0.65	1.00	0.98	0.11	1.00	0.98	0.18	1.00	0.98
mid-Co–B	0.68	1.00	0.98	0.83	1.00	0.98	0.014	1.00	0.51	0.045	1.00	0.77
mid-Co–Gn	0.41	1.00	0.98	0.66	1.00	0.98	0.025	1.00	0.54	0.027	1.00	0.55
mid-Go–Gn	0.40	1.00	0.98	0.68	1.00	0.98	0.069	1.00	0.81	0.061	1.00	0.78
mid-Co–mid-Go	0.90	1.00	0.98	0.75	1.00	0.98	0.81	1.00	0.98	0.86	1.00	0.98
ramus length/SN length	0.58	1.00	0.98	0.89	1.00	0.98	0.40	1.00	0.98	0.80	1.00	0.98
mandibular body length/SN length	0.42	1.00	0.98	0.62	1.00	0.98	0.94	1.00	0.99	0.44	1.00	0.98
ramus length/mandibular body length	0.99	1.00	0.99	0.52	1.00	0.98	0.41	1.00	0.98	0.45	1.00	0.98
ramus length/ANS-PNS	0.43	1.00	0.98	0.74	1.00	0.98	0.10	1.00	0.98	0.73	1.00	0.98
mid-Co–Gn/ANS–PNS	0.27	1.00	0.98	0.51	1.00	0.98	0.13	1.00	0.98	0.98	1.00	0.99
mandibular body length/ANS–PNS	0.31	1.00	0.98	0.33	1.00	0.98	0.23	1.00	0.98	0.75	1.00	0.98
SN length/Co–Co	0.54	1.00	0.98	0.48	1.00	0.98	0.089	1.00	0.94	0.44	1.00	0.98
ramus length/Co–Co	0.98	1.00	0.99	0.81	1.00	0.98	0.50	1.00	0.98	0.61	1.00	0.98
mandibular body length/Co–Co	0.91	1.00	0.98	0.33	1.00	0.98	0.051	1.00	0.77	0.12	1.00	0.98
ANS–PNS/Co–Co	0.49	1.00	0.98	0.88	1.00	0.98	0.022	1.00	0.54	0.49	1.00	0.98
Go–Go/Co–Co	0.99	1.00	0.99	0.55	1.00	0.98	0.010	1.00	0.49	0.20	1.00	0.98
mid-Co–mid-Go/Co–Co	0.74	1.00	0.98	0.59	1.00	0.98	0.71	1.00	0.98	0.87	1.00	0.98
mid-Go–Gn/Co–Co	0.73	1.00	0.98	0.49	1.00	0.98	0.11	1.00	0.98	0.16	1.00	0.98
mid-Co–B/Co–Co	0.99	1.00	0.99	0.64	1.00	0.98	0.069	1.00	0.81	0.20	1.00	0.98
mid-Go–B/Co–Co	0.90	1.00	0.98	0.48	1.00	0.98	0.17	1.00	0.98	0.36	1.00	0.98
mid-Co–Gn/Co–Co	0.77	1.00	0.98	0.51	1.00	0.98	0.087	1.00	0.93	0.14	1.00	0.98
SNA	0.62	1.00	0.98	0.46	1.00	0.98	0.64	1.00	0.98	0.80	1.00	0.98
SNB	0.16	1.00	0.98	0.76	1.00	0.98	0.88	1.00	0.98	0.25	1.00	0.98
ANB	0.18	1.00	0.98	0.39	1.00	0.98	0.35	1.00	0.98	0.077	1.00	0.86
FMA	0.97	1.00	0.99	0.93	1.00	0.99	0.40	1.00	0.98	0.57	1.00	0.98
S–N–Pog angle	0.20	1.00	0.98	0.35	1.00	0.98	0.69	1.00	0.98	0.15	1.00	0.98
S–N–Pog–SNB difference	0.88	1.00	0.98	0.060	1.00	0.78	0.50	1.00	0.98	0.28	1.00	0.98
Gonial angle (mid-Co–mid-Go–Gn)	0.93	1.00	0.99	0.79	1.00	0.98	0.99	1.00	0.99	0.97	1.00	0.99
mid-Go–Gn–B	0.33	1.00	0.98	0.86	1.00	0.98	0.72	1.00	0.98	0.41	1.00	0.98
mid-Go–Pog–B	0.43	1.00	0.98	0.81	1.00	0.98	0.67	1.00	0.98	0.47	1.00	0.98

Outcome variable	Japan (n = 199)			Korea (n = 79)			Egypt (n = 170)			Fixed-effect meta-analysis		
	p-value	Bonferroni P	BH-FDR P	*p*-value	Bonferroni P	BH-FDR P	*p*-value	Bonferroni P	BH-FDR P	*p*-value	Bonferroni P	BH-FDR P
mid-Go–Pog–Gn	0.56	1.00	0.98	0.64	1.00	0.98	0.73	1.00	0.98	0.66	1.00	0.98
GoR–Gn–GoL	0.81	1.00	0.98	0.26	1.00	0.98	0.50	1.00	0.98	0.87	1.00	0.98
ln (centroid size)	0.25	1.00	0.98	0.76	1.00	0.98	0.029	1.00	0.57	0.023	1.00	0.54
PC1	0.38	1.00	0.98	0.43	1.00	0.98	0.48	1.00	0.98	0.19	1.00	0.98
PC2	0.40	1.00	0.98	0.88	1.00	0.98	0.30	1.00	0.98	0.20	1.00	0.98
PC3	0.86	1.00	0.98	0.0047	1.00	0.49	0.12	1.00	0.98	0.79	1.00	0.98
PC4	0.90	1.00	0.98	0.97	1.00	0.99	0.62	1.00	0.98	0.70	1.00	0.98
PC5	0.053	1.00	0.77	0.99	1.00	0.99	0.26	1.00	0.98	0.46	1.00	0.98
PC6	0.83	1.00	0.98	0.91	1.00	0.98	0.46	1.00	0.98	0.78	1.00	0.98
PC7	0.10	1.00	0.98	0.32	1.00	0.98	0.28	1.00	0.98	0.37	1.00	0.98
PC8	0.79	1.00	0.98	0.83	1.00	0.98	0.013	1.00	0.51	0.24	1.00	0.98
PC9	0.63	1.00	0.98	0.0065	1.00	0.49	0.53	1.00	0.98	0.56	1.00	0.98
PC10	0.41	1.00	0.98	0.39	1.00	0.98	0.52	1.00	0.98	0.19	1.00	0.98
PC11	0.56	1.00	0.98	0.25	1.00	0.98	0.57	1.00	0.98	0.21	1.00	0.98
rs2242116												
Volume	0.83	1.00	0.98	0.97	1.00	0.99	0.60	1.00	0.98	0.87	1.00	0.98
SN length	0.85	1.00	0.98	0.72	1.00	0.98	0.063	1.00	0.78	0.25	1.00	0.98
ramus length	0.85	1.00	0.98	0.94	1.00	0.99	1.0	1.00	1.00	0.91	1.00	0.98
mandibular body length	0.35	1.00	0.98	0.48	1.00	0.98	0.11	1.00	0.98	0.056	1.00	0.78
ANS-PNS	0.43	1.00	0.98	0.59	1.00	0.98	0.052	1.00	0.77	0.79	1.00	0.98
intergonial width (Go-Go)	0.42	1.00	0.98	0.18	1.00	0.98	0.0067	1.00	0.49	0.11	1.00	0.98
intercondylar width (Co-Co)	0.53	1.00	0.98	0.48	1.00	0.98	0.81	1.00	0.98	0.74	1.00	0.98
mid-Go-B	0.87	1.00	0.98	0.65	1.00	0.98	0.67	1.00	0.98	0.60	1.00	0.98
mid-Co–B	0.74	1.00	0.98	0.83	1.00	0.98	0.33	1.00	0.98	0.34	1.00	0.98
mid-Co–Gn	0.35	1.00	0.98	0.66	1.00	0.98	0.31	1.00	0.98	0.15	1.00	0.98
mid-Go–Gn	0.33	1.00	0.98	0.68	1.00	0.98	0.45	1.00	0.98	0.20	1.00	0.98
mid-Co–mid-Go	0.77	1.00	0.98	0.75	1.00	0.98	0.86	1.00	0.98	0.83	1.00	0.98
ramus length/SN length	0.78	1.00	0.98	0.89	1.00	0.98	0.26	1.00	0.98	0.53	1.00	0.98
mandibular body length/SN length	0.41	1.00	0.98	0.62	1.00	0.98	0.82	1.00	0.98	0.56	1.00	0.98
ramus length/mandibular body length	0.72	1.00	0.98	0.52	1.00	0.98	0.37	1.00	0.98	0.30	1.00	0.98
ramus length/ANS-PNS	0.42	1.00	0.98	0.74	1.00	0.98	0.14	1.00	0.98	0.80	1.00	0.98

Outcome variable	Japan (n = 199)			Korea (n = 79)			Egypt (n = 170)			Fixed-effect meta-analysis		
	p-value	Bonferroni P	BH-FDR P	*p*-value	Bonferroni P	BH-FDR P	*p*-value	Bonferroni P	BH-FDR P	*p*-value	Bonferroni P	BH-FDR P
mid-Co–Gn/ANS–PNS	0.18	1.00	0.98	0.51	1.00	0.98	0.18	1.00	0.98	0.76	1.00	0.98
mandibular body length/ANS–PNS	0.17	1.00	0.98	0.33	1.00	0.98	0.35	1.00	0.98	0.47	1.00	0.98
SN length/Co–Co	0.56	1.00	0.98	0.48	1.00	0.98	0.32	1.00	0.98	0.69	1.00	0.98
ramus length/Co–Co	0.81	1.00	0.98	0.81	1.00	0.98	0.84	1.00	0.98	0.84	1.00	0.98
mandibular body length/Co–Co	0.88	1.00	0.98	0.33	1.00	0.98	0.34	1.00	0.98	0.32	1.00	0.98
ANS–PNS/Co–Co	0.34	1.00	0.98	0.88	1.00	0.98	0.16	1.00	0.98	0.99	1.00	0.99
Go–Go/Co–Co	0.80	1.00	0.98	0.55	1.00	0.98	0.016	1.00	0.51	0.17	1.00	0.98
mid-Co–mid-Go/Co–Co	0.55	1.00	0.98	0.59	1.00	0.98	0.74	1.00	0.98	0.65	1.00	0.98
mid-Go–Gn/Co–Co	0.77	1.00	0.98	0.49	1.00	0.98	0.68	1.00	0.98	0.49	1.00	0.98
mid-Co–B/Co–Co	0.84	1.00	0.98	0.64	1.00	0.98	0.62	1.00	0.98	0.74	1.00	0.98
mid-Go–B/Co–Co	0.70	1.00	0.98	0.48	1.00	0.98	0.89	1.00	0.98	0.97	1.00	0.99
mid-Co–Gn/Co–Co	0.83	1.00	0.98	0.51	1.00	0.98	0.61	1.00	0.98	0.48	1.00	0.98
SNA	0.75	1.00	0.98	0.46	1.00	0.98	0.078	1.00	0.86	0.27	1.00	0.98
SNB	0.15	1.00	0.98	0.76	1.00	0.98	0.25	1.00	0.98	0.78	1.00	0.98
ANB	0.11	1.00	0.98	0.39	1.00	0.98	0.33	1.00	0.98	0.051	1.00	0.77
FMA	0.78	1.00	0.98	0.93	1.00	0.99	0.40	1.00	0.98	0.44	1.00	0.98
S–N–Pog angle	0.17	1.00	0.98	0.35	1.00	0.98	0.41	1.00	0.98	0.53	1.00	0.98
S–N–Pog–SNB difference	0.95	1.00	0.99	0.060	1.00	0.78	0.42	1.00	0.98	0.22	1.00	0.98
Gonial angle (mid-Co–mid-Go–Gn)	0.82	1.00	0.98	0.79	1.00	0.98	0.72	1.00	0.98	0.85	1.00	0.98
mid-Go–Gn–B	0.15	1.00	0.98	0.86	1.00	0.98	0.65	1.00	0.98	0.23	1.00	0.98
mid-Go–Pog–B	0.26	1.00	0.98	0.81	1.00	0.98	0.66	1.00	0.98	0.33	1.00	0.98
mid-Go–Pog–Gn	0.33	1.00	0.98	0.64	1.00	0.98	0.98	1.00	0.99	0.62	1.00	0.98
GoR–Gn–GoL	0.98	1.00	0.99	0.26	1.00	0.98	0.14	1.00	0.98	0.61	1.00	0.98
ln (centroid size)	0.14	1.00	0.98	0.76	1.00	0.98	0.052	1.00	0.77	0.019	1.00	0.54
PC1	0.47	1.00	0.98	0.43	1.00	0.98	0.58	1.00	0.98	0.66	1.00	0.98
PC2	0.18	1.00	0.98	0.88	1.00	0.98	0.27	1.00	0.98	0.10	1.00	0.98
PC3	0.97	1.00	0.99	0.0047	1.00	0.49	0.29	1.00	0.98	0.53	1.00	0.98
PC4	0.61	1.00	0.98	0.97	1.00	0.99	0.36	1.00	0.98	0.37	1.00	0.98
PC5	0.031	1.00	0.57	0.99	1.00	0.99	0.90	1.00	0.98	0.13	1.00	0.98
PC6	0.72	1.00	0.98	0.91	1.00	0.98	0.21	1.00	0.98	0.60	1.00	0.98
PC7	0.13	1.00	0.98	0.32	1.00	0.98	0.36	1.00	0.98	0.37	1.00	0.98

Outcome variable	Japan (n = 199)			Korea (n = 79)			Egypt (n = 170)			Fixed-effect meta-analysis		
	p-value	Bonferroni P	BH-FDR P	*p*-value	Bonferroni P	BH-FDR P	*p*-value	Bonferroni P	BH-FDR P	*p*-value	Bonferroni P	BH-FDR P
PC8	0.84	1.00	0.98	0.83	1.00	0.98	0.0079	1.00	0.49	0.18	1.00	0.98
PC9	0.48	1.00	0.98	0.0065	1.00	0.49	0.74	1.00	0.98	0.57	1.00	0.98
PC10	0.56	1.00	0.98	0.39	1.00	0.98	0.60	1.00	0.98	0.62	1.00	0.98
PC11	0.53	1.00	0.98	0.25	1.00	0.98	0.57	1.00	0.98	0.20	1.00	0.98

Raw P values are two-sided and unadjusted. Bonferroni-adjusted and Benjamini-Hochberg false-discovery-rate (BH-FDR)-adjusted *p* values were calculated across the complete family of 408 secondary tests, comprising 51 outcomes, 2 SNPs, and 4 analysis sets (Japan, Korea, Egypt, and the fixed-effect meta-analysis). Cohort-specific regression models were adjusted for sex and age and, in the Japan-recruited cohort, additionally for imaging modality. Fixed-effect meta-analysis estimates were obtained using inverse-variance weighting. Genotypes were coded additively as 0, 1, or 2 according to the number of derived A alleles. No test remained statistically significant after either multiple-testing correction. ln(centroid size) was analyzed on the natural-log scale. PTH1R, parathyroid hormone 1 receptor; SNP, single-nucleotide polymorphism; BH-FDR, Benjamini-Hochberg false-discovery rate.

Table S8. Comparison of rs2242116 fixed-effect meta-analysis estimates between the original 448-participant sample and all 458 eligible participants.

SNP	Outcome	Original analysis (N=448)		Sensitivity analysis (N=458)			
		B (95% CI)	p-value	B (95% CI)	p-value	Bonferroni P	BH-FDR P
rs2242116	PC1	-0.0007 (-0.0039, 0.0025)	0.66	-0.0007 (-0.0038, 0.0025)	0.68	1	0.96
	PC2	0.0023 (-0.0004, 0.0051)	0.10	0.0020 (-0.0007, 0.0047)	0.15	1	0.96
	PC3	-0.0007 (-0.0029, 0.0015)	0.53	-0.0005 (-0.0027, 0.0018)	0.69	1	0.96
	PC4	0.0008 (-0.0010, 0.0027)	0.37	0.0009 (-0.0009, 0.0027)	0.33	1	0.96
	PC5	-0.0013 (-0.0029, 0.0004)	0.13	-0.0013 (-0.0029, 0.0003)	0.11	1	0.96
	PC6	0.0004 (-0.0011, 0.0020)	0.60	0.0003 (-0.0012, 0.0019)	0.68	1	0.96
	PC7	0.0007 (-0.0008, 0.0021)	0.37	0.0007 (-0.0008, 0.0022)	0.36	1	0.96
	PC8	0.0008 (-0.0004, 0.0020)	0.18	0.0009 (-0.0003, 0.0021)	0.15	1	0.96
	PC9	0.0004 (-0.0009, 0.0017)	0.57	0.0004 (-0.0009, 0.0017)	0.55	1	0.96

SNP	Outcome	Original analysis (N=448)		Sensitivity analysis (N=458)			
		B (95% CI)	p-value	B (95% CI)	p-value	Bonferroni P	BH-FDR P
	PC10	0.0003 (-0.0009, 0.0014)	0.62	0.0004 (-0.0008, 0.0015)	0.54	1	0.96
	PC11	0.0007 (-0.0004, 0.0017)	0.20	0.0007 (-0.0003, 0.0018)	0.17	1	0.96
	ln (centroid size)	-0.0054 (-0.0100, -0.0009)	0.019*	-0.0052 (-0.0097, -0.0007)	0.022*	1	0.74
	Volume	67.0939 (-726.9523, 861.1401)	0.87	64.2176 (-717.2177, 845.6529)	0.87	1	0.96
	SN length	-0.2288 (-0.6197, 0.1620)	0.25	-0.2124 (-0.5988, 0.1740)	0.28	1	0.96
	ramus length	-0.0314 (-0.5939, 0.5311)	0.91	-0.0531 (-0.6082, 0.5020)	0.85	1	0.96
	mandibular body length	-0.5166 (-1.0473, 0.0141)	0.06	-0.5070 (-1.0294, 0.0154)	0.06	1	0.96
	ANS-PNS	-0.0587 (-0.4929, 0.3756)	0.79	-0.0638 (-0.4926, 0.3649)	0.77	1	0.96
	intergonial width (Go-Go)	-0.6035 (-1.3495, 0.1425)	0.11	-0.5514 (-1.2886, 0.1857)	0.14	1	0.96
	intercondylar width (Co-Co)	-0.1196 (-0.8194, 0.5803)	0.74	-0.0608 (-0.7546, 0.6331)	0.86	1	0.96
	mid-Go-B	-0.1452 (-0.6914, 0.4009)	0.60	-0.1721 (-0.7096, 0.3654)	0.53	1	0.96
	mid-Co-B	-0.3315 (-1.0119, 0.3488)	0.34	-0.2710 (-0.9419, 0.3999)	0.43	1	0.96
	mid-Co-Gn	-0.5470 (-1.2862, 0.1923)	0.15	-0.5113 (-1.2409, 0.2183)	0.17	1	0.96
	mid-Go-Gn	-0.3827 (-0.9644, 0.1991)	0.20	-0.4098 (-0.9838, 0.1641)	0.16	1	0.96
	mid-Co-mid-Go	0.0652 (-0.5239, 0.6543)	0.83	0.0824 (-0.4978, 0.6626)	0.78	1	0.96
	ramus length/SN length	0.0031 (-0.0065, 0.0127)	0.53	0.0025 (-0.0069, 0.0120)	0.60	1	0.96
	mandibular body length/SN length	-0.0029 (-0.0128, 0.0069)	0.56	-0.0031 (-0.0128, 0.0066)	0.53	1	0.96

SNP	Outcome	Original analysis (N=448)		Sensitivity analysis (N=458)			
		B (95% CI)	p-value	B (95% CI)	p-value	Bonferroni P	BH-FDR P
	ramus length/mandibular body length	0.0034 (-0.0030, 0.0099)	0.30	0.0030 (-0.0034, 0.0094)	0.36	1	0.96
	ramus length/ANS-PNS	0.0019 (-0.0127, 0.0166)	0.80	0.0018 (-0.0127, 0.0162)	0.81	1	0.96
	mid-Co-Gn/ANS-PNS	-0.0040 (-0.0291, 0.0211)	0.76	-0.0046 (-0.0296, 0.0205)	0.72	1	0.96
	mandibular body length/ANS-PNS	-0.0062 (-0.0230, 0.0106)	0.47	-0.0058 (-0.0224, 0.0109)	0.50	1	0.96
	SN length/Co-Co	-0.0011 (-0.0067, 0.0044)	0.69	-0.0013 (-0.0068, 0.0042)	0.65	1	0.96
	ramus length/Co-Co	0.0007 (-0.0057, 0.0070)	0.84	0.0001 (-0.0062, 0.0064)	0.97	1	1.00
	mandibular body length/Co-Co	-0.0035 (-0.0103, 0.0034)	0.32	-0.0039 (-0.0106, 0.0028)	0.26	1	0.96
	ANS-PNS/Co-Co	-0.0000 (-0.0053, 0.0052)	0.99	-0.0003 (-0.0055, 0.0049)	0.92	1	0.98
	Go-Go/Co-Co	-0.0054 (-0.0131, 0.0024)	0.17	-0.0055 (-0.0132, 0.0022)	0.16	1	0.96
	mid-Co-mid-Go/Co-Co	0.0016 (-0.0051, 0.0083)	0.65	0.0013 (-0.0053, 0.0080)	0.69	1	0.96
	mid-Go-Gn/Co-Co	-0.0025 (-0.0096, 0.0046)	0.49	-0.0032 (-0.0102, 0.0038)	0.37	1	0.96
	mid-Co-B/Co-Co	-0.0016 (-0.0113, 0.0081)	0.74	-0.0018 (-0.0113, 0.0078)	0.72	1	0.96
	mid-Go-B/Co-Co	-0.0001 (-0.0070, 0.0067)	0.97	-0.0008 (-0.0075, 0.0060)	0.82	1	0.96
	mid-Co-Gn/Co-Co	-0.0037 (-0.0140, 0.0066)	0.48	-0.0041 (-0.0143, 0.0061)	0.43	1	0.96
	SNA	0.2705 (-0.2077, 0.7487)	0.27	0.2534 (-0.2210, 0.7278)	0.30	1	0.96
	SNB	-0.0803 (-0.6542, 0.4936)	0.78	-0.0627 (-0.6313, 0.5059)	0.83	1	0.96
	ANB	0.4099 (-0.0010, 0.8209)	0.05	0.3606 (-0.0484, 0.7695)	0.08	1	0.96

SNP	Outcome	Original analysis (N=448)		Sensitivity analysis (N=458)			
		B (95% CI)	p-value	B (95% CI)	p-value	Bonferroni P	BH-FDR P
	FMA	-0.3086 (-1.0840, 0.4668)	0.44	-0.2731 (-1.0430, 0.4968)	0.49	1	0.96
	S-N-Pog angle	-0.1908 (-0.7808, 0.3992)	0.53	-0.1772 (-0.7628, 0.4083)	0.55	1	0.96
	S-N-Pog-SNB difference	-0.1501 (-0.3907, 0.0905)	0.22	-0.1605 (-0.4017, 0.0808)	0.19	1	0.96
	Gonial angle (mid-Co-mid-Go-Gn)	0.0628 (-0.5862, 0.7119)	0.85	0.1397 (-0.5022, 0.7816)	0.67	1	0.96
	mid-Go-Gn-B	0.3991 (-0.2545, 1.0526)	0.23	0.3851 (-0.2659, 1.0362)	0.25	1	0.96
	mid-Go-Pog-B	0.3482 (-0.3531, 1.0495)	0.33	0.3322 (-0.3676, 1.0320)	0.35	1	0.96
	mid-Go-Pog-Gn	-0.2448 (-1.2032, 0.7136)	0.62	-0.2440 (-1.1873, 0.6992)	0.61	1	0.96
	GoR-Gn-GoL	-0.1489 (-0.7232, 0.4254)	0.61	-0.1019 (-0.6693, 0.4656)	0.72	1	0.96

Values are presented as regression coefficients (B), unadjusted 95% confidence intervals (CIs), and raw P values unless otherwise indicated. B represents the estimated change per additional A allele. Cohort-specific models adjusted for sex and age, and additionally for imaging modality in Japan. Estimates were combined using inverse-variance fixed-effect meta-analysis. Sensitivity-analysis Bonferroni and BH-FDR adjustments were applied across 204 tests, comprising 51 outcomes and four analysis sets (the three cohorts and the meta-analysis), although only meta-analysis results are displayed here. An asterisk indicates nominal raw $P < 0.05$. No sensitivity association remained significant after either correction. ln(centroid size) was analyzed on the natural-log scale. BH-FDR, Benjamini–Hochberg false-discovery rate; PC, principal component; SNP, single-nucleotide polymorphism.
